# Establishment, characterization, and biobanking of 36 pancreatic cancer organoids: prediction of metastasis in resectable pancreatic cancer

**DOI:** 10.1007/s13402-024-00939-5

**Published:** 2024-04-15

**Authors:** Soon-Chan Kim, Ha-Young Seo, Ja-Oh Lee, Ju Eun Maeng, Young-Kyoung Shin, Sang Hyub Lee, Jin-Young Jang, Ja-Lok Ku

**Affiliations:** 1 Korean Cell Line Bank, Laboratory of Cell Biology, Cancer Research Institute, Seoul National University College of Medicine, 101, Daehak-ro, Jongno-gu, Seoul, 03080 Korea; 2 Ischemic/Hypoxic Disease Institute, Seoul National University College of Medicine, Seoul, Korea; 3 Department of Internal Medicine and Liver Research Institute, Seoul National University Hospital, Seoul National University College of Medicine, 103 Daehak-ro, Jongno-gu, Seoul, 03080 Korea; 4 Department of Surgery, Seoul National University College of Medicine, 103, Daehak-ro, Jongno-gu, Seoul, 03080 South Korea; 5 Department of Biomedical Sciences, Seoul National University College of Medicine, Seoul, Korea

**Keywords:** Pancreatic adenocarcinoma, Metastasis, Prediction model, Organoid, Biobank

## Abstract

**Purpose:**

Early dissemination of primary pancreatic ductal adenocarcinoma (PDAC) is the main cause of dismal prognosis as it highly limits possible treatment options. A number of PDAC patients experience distant metastasis even after treatment due to the metastatic clones. We aimed to demonstrate the molecular architecture of borderline resectable PDAC manifests cancer dissemination of PDAC.

**Methods:**

Here, 36 organoids isolated from primary tumor masses of PDAC patients with diverse metastatic statues are presented. Whole-exome sequencing and RNA sequencing were performed and drug responses to clinically relevant 18 compounds were assessed.

**Results:**

Our results revealed that borderline resectable PDAC organoids exhibited distinct patterns according to their metastatic potency highlighted by multiple genetic and transcriptional factors and strong variances in drug responses.

**Conclusions:**

These data suggest that the presence of metastatic PDAC can be identified by integrating molecular compositions and drug responses of borderline resectable PDAC.

**Supplementary Information:**

The online version contains supplementary material available at 10.1007/s13402-024-00939-5.

## Introduction

Pancreatic ductal adenocarcinoma (PDAC) is one of the most aggressive malignancies with a propensity for early metastatic spread [1]. Approximately 80% of PDAC patients are diagnosed at an advanced stage with a dismal prognosis [2, 3]. Advances in sequencing techniques have enabled identification of a few molecular subtypes and suggested treatment options for certain types of PDACs in accordance with genomic and transcriptomic profiles [4]. Nevertheless, molecular characteristics of metastatic PDAC remain largely unclear, partially due to low assessment of surgical specimens of metastasized PDACs because many clinical practice guidelines designate metastatic PDACs as typically unresectable [5–7]. Reflecting its poor accessibility, prior large cohort studies using PDACs consisted of only subtle portion of metastatic samples [8–11]. Accordingly, pre-clinical models for validating actionable molecular targets of metastatic PDACs are very few in number as well [12].

Researchers studying metastatic PDACs have mostly focused on molecular profiles between metastatic deposits and early-stage disease in the pancreas as in many other solid tumors [13]. This approach has revealed that immunological and transcriptional alterations including cell cycle signaling, stem cell signaling and microenvironment remodeling are likely to be associated with dissemination of pancreatic tumor cells [14]. Genetic aberrations such as increasing *KRAS* mutant population have also been identified to facilitate metastasis [15]. Nevertheless, unlike other solid cancers, metastasis of PDAC can take place during initial progression of tumor, even prior to mass formation, suggesting that the molecular architecture of primary tumor might account for early dissemination of tumor cells [16–18]. Indeed, primary pancreatic tumor often metastasizes even after treatment [19, 20]. Therefore, distinguishing risk groups for high metastatic propensity based on molecular compositions of the primary tumor can benefit PDAC patients by providing clues to prevent unwanted progression. To comprehend molecular discrepancy of primary PDACs with potential metastatic capability, we established PDAC organoids isolated from primary tumor masses of 36 patients with diverse metastatic statuses and performed multi-omics and pharmacologic analysis. We also integrated actionable molecules in metastatic clones as predictive markers of targeted therapies in patients with metastatic progression using organoid models.

## Results

### Pancreatic tumor organoids from 36 patients recapitulate morphologic features of the original tumor

We successfully established a living biobank consisting of 36 organoids from primary pancreatic adenocarcinoma (PDAC) patients with various metastasis statuses. Twenty-two patients exhibited distant metastases in the liver (*n* = 14), lungs (*n* = 4), multiple sites (*n* = 2), and lymph nodes (*n* = 2). Among them, seven patients were initially diagnosed with borderline or resectable PDAC that progressed to the metastatic stage after treatment. We followed patients for a maximum of 24 months. Thirteen patients presented no metastasis during the follow-up period. Clinicopathological information including treatment regimen of each patient is summarized in Table 1. Fingerprinting analysis validated that all organoids were unique without cross-contamination (Supplementary Table 1). Most organoids were stably cultivated after consecutive passaging of 2 or 3 times, and cryopreserved at every passage.

**Table 1:** Clinicopathological information of 36 pancreatic tumor patients

No	Sample Name	Sex	Age	Location	Size (cm)	Stage	Treatment	Regimen	(Neo)-Adjuvant Treatment	Metastastic status
1	SNU-2543-TO	M	69	Tail	3.4	Resectable	Surgery	Distal pancreatectomy	CCRT ^c)^ with 5-FU	metastasis_liver
2	SNU-3898-TO	F	61	Body	1.7	Borderline	Surgery	Distal pancreatectomy	CCRT with gemcitabine -> operation -> gemcitabine	recur
3	SNU-3912-TO	F	58	Head	2.3	Resectable	Surgery	Whipple’s op	Palliative FOLFIRINOX	metastasis_lymph node
4	SNU-3923-TO	M	69	Head	4	Resectable	Surgery	PPPD ^a)^		no
5	SNU-3926-TO	F	52	Head	2.3	Borderline	Surgery	Total pancreatectomy	mFOLFIRINOX ^d)^ -> operation -> adjuvent gemcitabine	no
6	SNU-3947-TO	F	64	Tail	7.2	Metastatic	Chemotherapy	Gemcitabine/nab-paclitaxel	-	metastasis_multi
7	SNU-3997-TO	M	54	Head	3	Resectable	Surgery	Whipple’s op	Palliative FOLFIRINOX	metastasis_liver
8	SNU-4158-TO	F	54	Body	3.5	Metastatic	Chemotherapy	FOLFIRINOX ^b)^	-	metastasis_liver
9	SNU-4192-TO	F	56	Body	7.7	Metastatic	Chemotherapy	FOLFIRINOX	-	metastasis_liver
10	SNU-4206-TO	F	81	Head	2.1	Locally advanced	Chemotherapy	FOLFIRINOX	-	no
11	SNU-4208-TO	M	45	Body	4.3	Metastatic	Chemotherapy	FOLFIRINOX	-	metastasis_lung
12	SNU-4242-TO	M	74	Tail	4.4	Resectable	Surgery	Distal pancreatectomy	FOLFIRINOX	no
13	SNU-4243-TO	M	63	Tail	3.3	Resectable	Surgery	Distal pancreatectomy	5-FU/leucovorin	no
14	SNU-4305-TO	F	59	Head	2.5	Locally advanced	Chemotherapy	FOLFIRINOX	-	no
15	SNU-4309-TO	F	17	Tail	3.6	Metastatic	Chemotherapy	FOLFIRINOX	-	metastasis_liver
16	SNU-4340-TO	M	66	Tail	6.8	Metastatic	Chemotherapy	FOLFIRINOX	-	metastasis_liver
17	SNU-4354-TO	F	64	Body	2.4	Locally advanced	Chemotherapy	FOLFIRINOX	-	no
18	SNU-4365-TO		55	Head	2.2	Metastatic	Chemotherapy	FOLFIRINOX	-	metastasis_liver
19	SNU-4378-TO	F	69	Head	5	Locally advanced	Chemotherapy	FOLFIRINOX	-	no
20	SNU-4425-TO	F	60	Head	3.8	Metastatic	Chemotherapy	FOLFIRINOX	-	metastasis_lymph node
21	SNU-4457-TO	M	58	Head	2.3	Metastatic	Chemotherapy	FOLFIRINOX	-	metastasis_liver
22	SNU-4461-TO	M	67	Body	8	Metastatic	Chemotherapy	FOLFIRINOX	-	metastasis_liver_lymph node
23	SNU-4482-TO	M	65	Tail	3.7	Resectable	Surgery	Total pancreatectomy	gemcitabine and erotinib	metastasis_liver
24	SNU-4525-TO	M	56	Tail	4	Resectable	Surgery	Distal pancreatectomy	CCRT with 5-FU	no
25	SNU-4557-TO	F	63	Body	4.5	Locally advanced	Chemotherapy	FOLFIRINOX	-	no
26	SNU-4607-TO	F	57	Tail	5.5	Metastatic	Chemotherapy	FOLFIRINOX	-	metastasis_lung
27	SNU-4779-TO	F	62	Body	4	Metastatic	Chemotherapy	FOLFIRINOX	-	metastasis_lung
28	SNU-4837-TO	M	67	Body	5.4	Locally advanced	Chemotherapy	FOLFIRINOX		no
29	SNU-4863-TO	F	69	Head	4	Borderline	Surgery	Whipple’s op	Neo FOLFIRINOX	metastasis_liver
30	SNU-4871-TO	F	58	Head	1.6	Borderline	Surgery	PPPD	Neo FOLFIRINOX -> operation -> adjuvent CCRT with 5-FU	metastasis_lung
31	SNU-4874-TO	F	54	Head	3	Borderline	Surgery	Whipple’s op	Neo FOLFIRINOX -> operation -> adjuvent FOLFIRINOX	no
32	SNU-4893-TO	M	69	Tail	5.5	Metastatic	Chemotherapy	FOLFIRINOX	-	metastasis_liver_lymph node
33	SNU-4894-TO	M	61	Head	4.1	Metastatic	Chemotherapy	FOLFIRINOX	-	metastasis_liver_lymph node
34	SNU-5177-TO	F	57	Head	3.8	Borderline	Surgery	PPPD	Neo FOLFIRINOX -> operation -> adjuvent gemcitabine	metastasis_liver
35	SNU-5420-TO	M	56	Tail	5.6	Metastatic	Chemotherapy	FOLFIRINOX	-	metastasis_multi
36	SNU-5577-TO	M	43	Head	3.2	Locally advanced	Chemotherapy	FOLFIRINOX		no

^a)^PPPD indicates pylorus preserving pancreaticoduodenectomy; ^b)^FOLFIRINOX indicates fluorouracil, leucovorin, irinotecan, and oxaliplatin; ^c)^CCRT indicates concurrent chemoradiation therapy; ^d)^mFOLFIRINOX indicates modified FOLFIRINOX

**Fig. 1:**
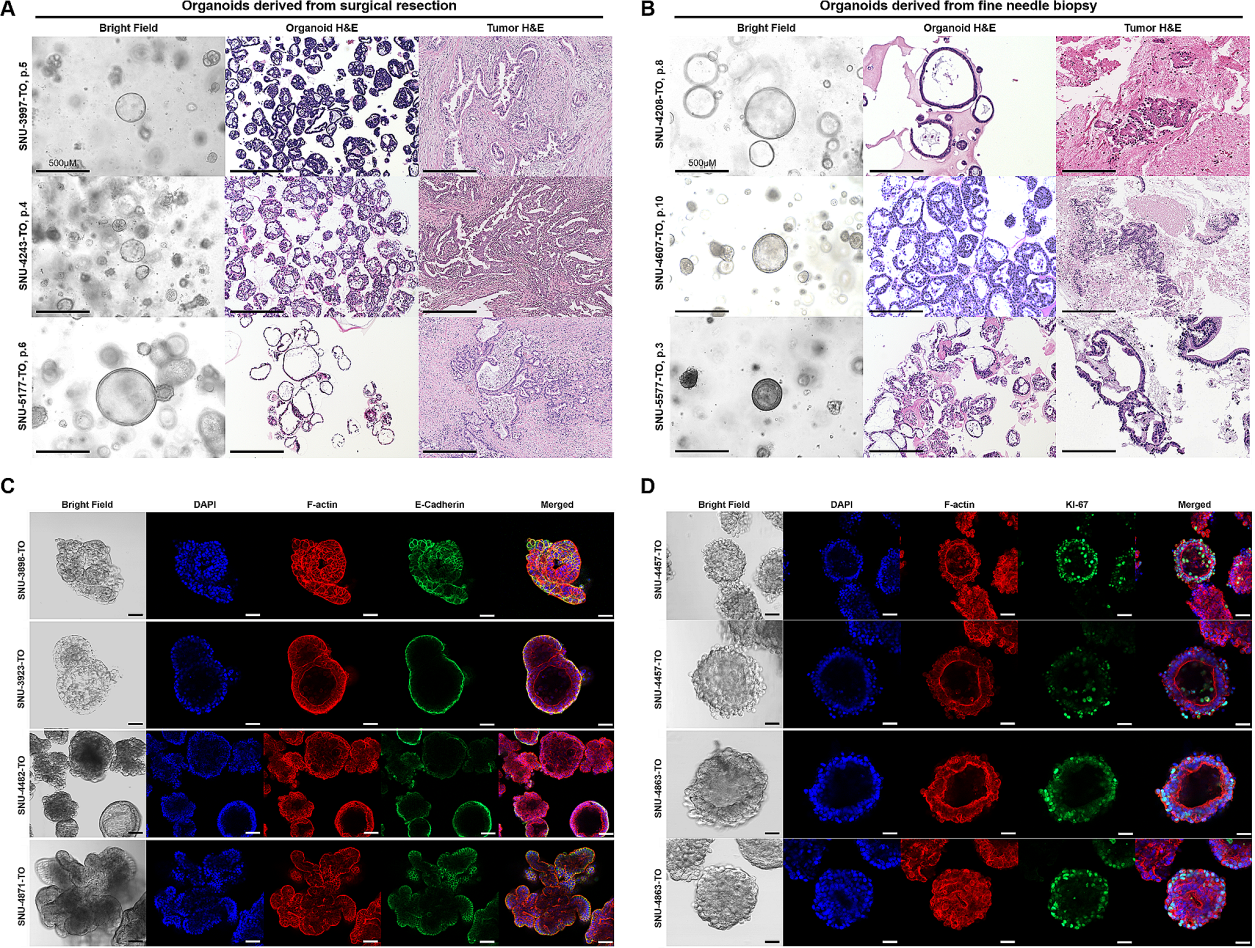
Histopathological Characterization of Pancreatic Ductal Adenocarcinoma Organoids. See also Supplementary Figs. 1, 2. Histological resemblance of PDAC organoids originated from **(A)** the surgical resection and **(B)** the fine needle aspiration biopsy. Scale bar = 500 µM. Most organoids showed thin-walled cystic structure with one or more lumen. Tumors devoid of the lumen formed compact organoids. Hematoxylin-eosin (H&E) staining of formalin-fixed paraffin-embedded (FFPE) organoid sections confirmed that most organoids resembled histological structures of the original tumors. Most organoid lines displayed mixed solid, pebble-like morphologies and cystic, balloon-like morphologies. Scale bar = 500 µM **(C)** Expressional patterns of E-cadherin in accordance with varying metastatic potency. Scale bar = 50 µM **(D)** Expressional patterns of KI-67 according to heterogeneous morphologies of PDAC organoids. For immunocytochemistry, organoids in their passages 1–3 were used. Scale bar = 50 µM

Majority of established organoids showed thin-walled cystic structure with one or more lumen. Tumors devoid of the lumen formed compact organoids without the lumen. Hematoxylin-eosin (H&E) staining of formalin-fixed paraffin-embedded (FFPE) organoid sections revealed that most organoids resembled histological structures of original tumors regardless of the isolation method. (Figs. 1A, B and Supplementary Fig. 1A, B). Although loss of E-cadherin is known to initiate dissemination of tumor cells in many tumors [21], it was observed only in a subset of metastatic PDAC [22]. We selected four representative organoids to validate the expressional pattern of E-cadherin in accordance with different metastatic statuses (Fig. 1C). SNU-3898-TO patient experienced recurrence seven months after distal pancreatectomy and the organoid showed a loosely aggregated morphology without distinct luminal layers. E-cadherin was clearly expressed in most cell junctions with varying degrees. Co-localization of E-cadherin with actin cytoskeleton was observed. SNU-3923-TO patient had no distant metastasis after pylorus preserving pancreaticoduodenectomy (PPPD) surgery. The organoid had compact forms with lumens. E-cadherin was clearly stained along with the outmost lining of the luminal layer. SNU-4482 patient had liver metastasis after total pancreatectomy. The organoid derived from this patient displayed heterogeneous morphologies with mixed grape-like shapes and compact structures showing clear lumens. E-cadherin was sporadically expressed along with the outer region of the organoid at different degrees. SNU-4871-TO patient developed metastasis in lungs and pleura after PPPD surgery, with derived organoids showing crypt-like structures, and E-cadherin localized in most cell junctions. Overall, in line with previous reports, cellular expression of E-cadherin in metastatic PDAC did not have specific patterns, lowering its value as a predictive marker for metastatic potency. We further correlated the level of E-cadherin expression with the metastatic status of the organoids as shown in Fig. 1C. SNU-3898-TO (recurrence) and SNU-4871-TO (lung metastasis) exhibited higher E-cadherin expression compared to SNU-3923-TO (non-metastatic), while SNU-4482-TO (liver metastasis) demonstrated lower E-cadherin expression relative to SNU-3923-TO (Supplementary Table 2A and Supplementary Fig. 2A).

KI-67 is present in actively proliferating tumor cells. It is a predictive marker for various types of pancreatic cancer including neuroendocrine tumors [23] and adenocarcinoma [24, 25]. Tumor cells at the inner part of compact organoids with grape-like shapes may experience less oxygen and nutrient gradients than those at the outer region, which might affect proliferation. We confirmed stable expression of KI-67 in the inner region of SNU-4457-TO with grape-like shape and clear luminal layers in two different passages. SNU-4863-TO displayed heterogeneous populations of compact organoids and cystic organoids within an equivalent linage. Both compact and cystic organoids actively expressed KI-67, potentially indication that structure variations of PDAC organoid would rarely affect proliferation of tumor cells (Fig. 1D, Supplementary Table 2B, Supplementary Fig. 2B). Nevertheless, it should be noted that the KI-67 indicates whether cells are out of the quiescent state, but it does not differentiate between different rates of cell cycle progression or cell cycle lengths among different populations. Structural variations in organoids could still potentially impact factors such as nutrient and oxygen diffusion, which in turn could affect cellular metabolism and proliferation rates differentially across the organoid. Overall, these data demonstrate that we have successfully established PDAC organoids recapitulating original morphological features.

### Mutational profiling of metastatic and non-metastatic PDAC organoids identifies VAF-dependent metastatic potential

We then accessed mutational profiles of PDAC organoids using whole-exome sequencing (WES). Among the 36 established pancreatic organoids, SNU-2543-TO was excluded from the WES analysis due to low DNA integrity. It has been repeatedly reported that organoids can recapitulate genetic alterations of the original tumor [26–28]. Since the tumor mass obtained from surgical resection and fine needle aspiration biopsy was limited, we selected eight tumor tissue-organoid pairs to validate that our PDAC organoids retained most mutations of the original tumor. Overall, nearly 90% of mutations were shared between tumor tissues and organoids (Supplementary Fig. 3A). We also compared variant allele frequencies (VAFs) of each mutation to confirm clonal composition was maintained in organoids during consecutive cultures. All eight pairs displayed correlation coefficient (R) > 0.85, suggesting that the mutational population of the original tumor tissue was mostly retained in organoids (Supplementary Fig. 3B). Overall scatter patterns of VAFs indicated that mutations were getting enriched in organoid samples, implying that continuous passaging might put selective force to prefer certain mutational clones (Supplementary Fig. 3C).

**Fig. 2:**
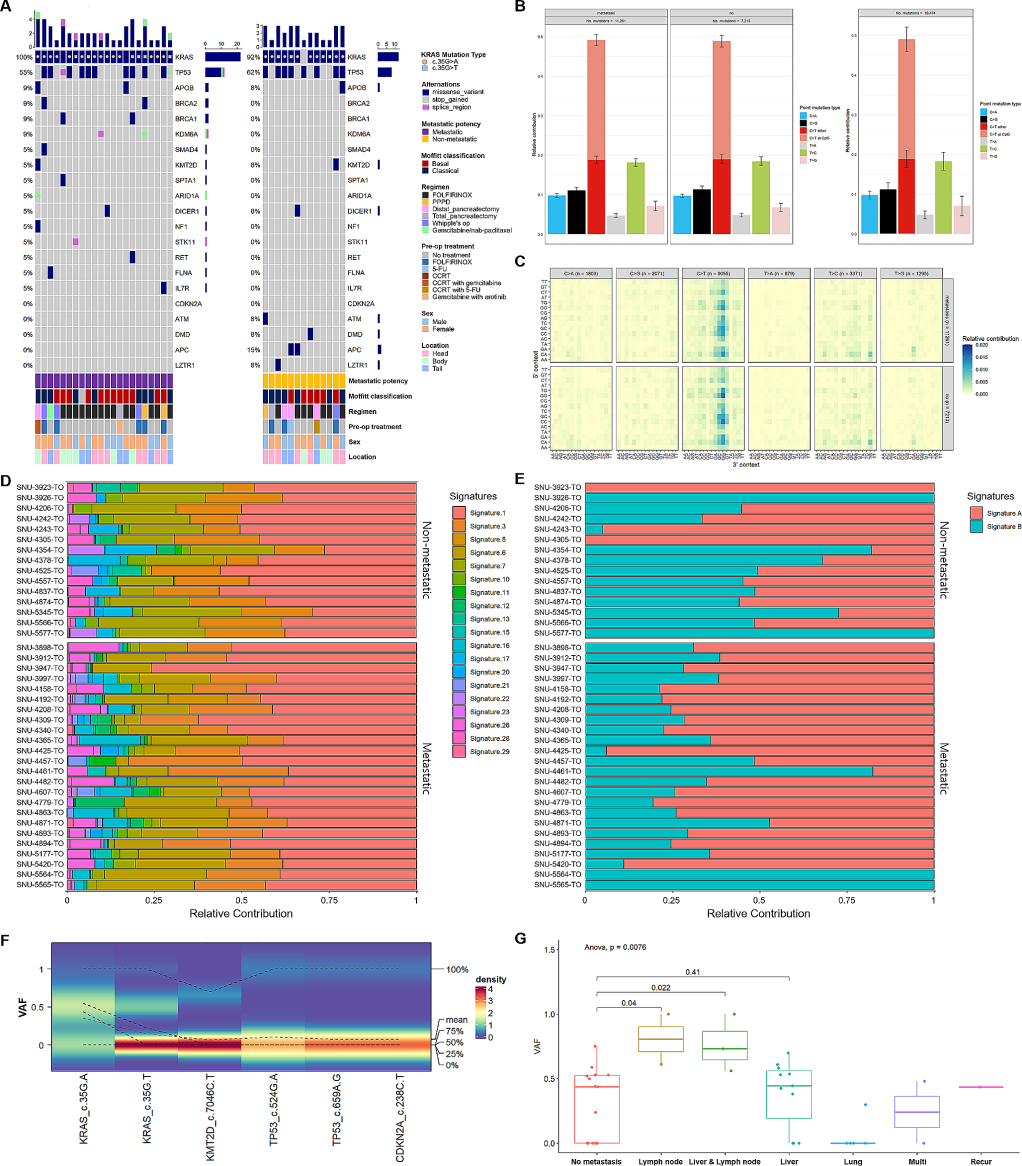
Mutational Fraction rather than Mutational Pattern Designates Metastatic Potency. See also Supplementary Figs. 3, 4 and Supplementary Table 3. **(A)** Representative genes that are mostly mutated in PDAC were compared between organoids groups with metastatic potency (MP, *n* = 22) and non-metastatic potency (NMP, *n* = 13). Mutational frequency was calculated in both groups. The number of mutation per each organoid and gene was indicated with bar plots. Each mutation type as well as clinicopathological information are marked with representative colors. The passages of organoids used for whole exome sequencing analysis were p2– p5. **(B)** Relative contribution of the point mutation types was estimated in both groups. Each mutation type was indicated with representative colors. **(C)** Relative contribution of the point mutation types was detailed according to the 3’ and 5’ context. The total number of six representative point mutation type was specified. **(D)** Relative contribution of mutational signatures based on pre-designated repertoire of mutational processes was compared between organoids groups with MP and NMP. Each signature was indicated with representative colors. **(E)** Relative contribution of two mutational signatures extracted from the point mutation profiles of our PDAC cohort was compared between organoids groups with MP and NMP. Two signatures were specified with representative colors. **(F)** Density plot presents the variant allele frequencies (VAFs) for five representative point mutations. When VAFs are skewed towards 0, as observed with the KMT2D c.7046 C > T mutation, the density of VAF is high at 0 and is highlighted in red. Conversely, when VAFs are not skewed and are more evenly distributed, as with the KRAS c.35G > A mutation, the density is lower and is highlighted in green. **(G)** Dispersion of VAF *KRAS* c.35G > A according to metastasis sites was compared. *P* value from ANOVA was specified above the bar plot and *p* values from t-test were designated between compared sites

Twenty-one genes including *KRAS*, *TP53*, *SMAD4*, and *CDKN2A* frequently involved in tumorigenesis and metastasis of pancreatic tumor [1, 29] were compared between organoid groups with metastatic potency (MP, *n* = 22) and non-metastatic potency (NMP, *n* = 13) (Fig. 2A). Since DNAs from matched blood or normal tissue were unavailable when WES was performed, we referred to the Clinvar database (https://www.ncbi.nlm.nih.gov/clinvar) to exclude benign mutations. Mutational pathogenicity of representative driver genes was manually inspected. All marked mutations for calculating the frequency of mutated genes in Fig. 2A and Supplementary Table 3A were previously reported as “Pathogenic” or “Conflicting interpretations of pathogenicity” in the Clinvar database.

Primary PDAC organoids with MP had analogous number of driver mutations per organoid compared to NMP PDAC organoids (Fig. 2A, Supplementary Table 3A). In parallel with previous findings [30, 31], majority of PDAC organoids had mutations in *KRAS* and *TP53* genes regardless of their metastatic potency. Most of *KRAS* mutations were glycine substitutions in codon 35: c.35G > A (*n* = 22) and c.35G > T (*n* = 10) followed by c.34G > C (*n* = 1), c.183A > C (*n* = 1) and c.340G > A (*n* = 1). We also compared mutational signatures between MP and NMP organoid groups. The composition of point mutation types was highly analogous, having predominant C to T transition at CpG site in both groups (Fig. 2B and Supplementary Table 3B). The relative contribution of each point mutation type remained highly similar even after specifying 5’ and 3’ context (Fig. 2C and Supplementary Table 3 C), suggesting that specific type of point mutation could rarely separate MP from NMP groups in our PDAC cohort. We also analyzed other mutation types such as deletion and insertion (Supplementary Fig. 4A). No specific pattern stood out in parallel with previous analysis of point mutation types. We then assigned mutational composition of each PDAC organoids to pre-designated repertoire of mutational processes [32]. Majority of organoids showed large portion of signature 1, signature 3, and signature 7. However, a distinct mutational pattern between MP and NMP groups was not detected (Fig. 2D). We also extracted two representative mutational signatures by applying non-negative matrix factorization (NMF) based on point mutation profiles of our PDAC cohort (Fig. 2E), which revealed enriched population of A[T > G]G mutation type (Supplementary Fig. 4B). However, the combination of point mutation types could not explain characteristics of metastatic clones in the primary PDAC.

It has been reported that varying amino acid substitutions in *KRAS* can result in different aberrations in multiple biological processes of tumor [33, 34]. We compared VAFs of two different *KRAS* mutation types in codon 35 between MP and NMP organoids groups (Supplementary Table 3D). Majority of c.35G > T mutation had VAFs of ∼ 0.5 or 1 regardless of the metastatic potency, whereas VAFs of c.35G > A displayed more sporadic patterns especially in MP groups (Fig. 2F). In our findings, a number of PDAC organoids displaying metastatic potency (MP) presented with variant allele frequencies (VAFs) exceeding 0.5 for the c.35G > A mutation in KRAS, suggesting that this mutation may play a prominent role within the tumor’s clonal hierarchy at the time of surgical resection. It’s important to consider, however, that elevated VAFs could also be indicative of genome duplication events or an increase in KRAS dosage, both of which are known to contribute to the aggressive behavior of tumor biology. Therefore, while our data might suggest a potential association between the c.35G > A mutation and the metastatic capacity of PDAC, further investigation is required to fully understand the implications of KRAS mutations, including c.35G > A and c.35G > T, in the context of PDAC progression and metastasis. Few other pathogenic mutations such as *KMT2D* c.7046 C > T, *TP53* c.524G > A, and *TP53* c.659 A > G had VAFs of ∼ 0.5 or 1. There were no feasible differences in accordance with metastatic potency, suggesting that those mutations took place at the initial progression of pancreatic tumor regardless of metastatic potency. Analysis of variance (ANOVA) validated that lymph node metastasized organoids had significantly higher site-specific VAFs of *KRAS* c.35G > A mutation than NMP organoids (Fig. 2G).

### Expressional patterns of metastatic and non-metastatic PDAC organoids reveal distinct pathway activation

**Fig. 3:**
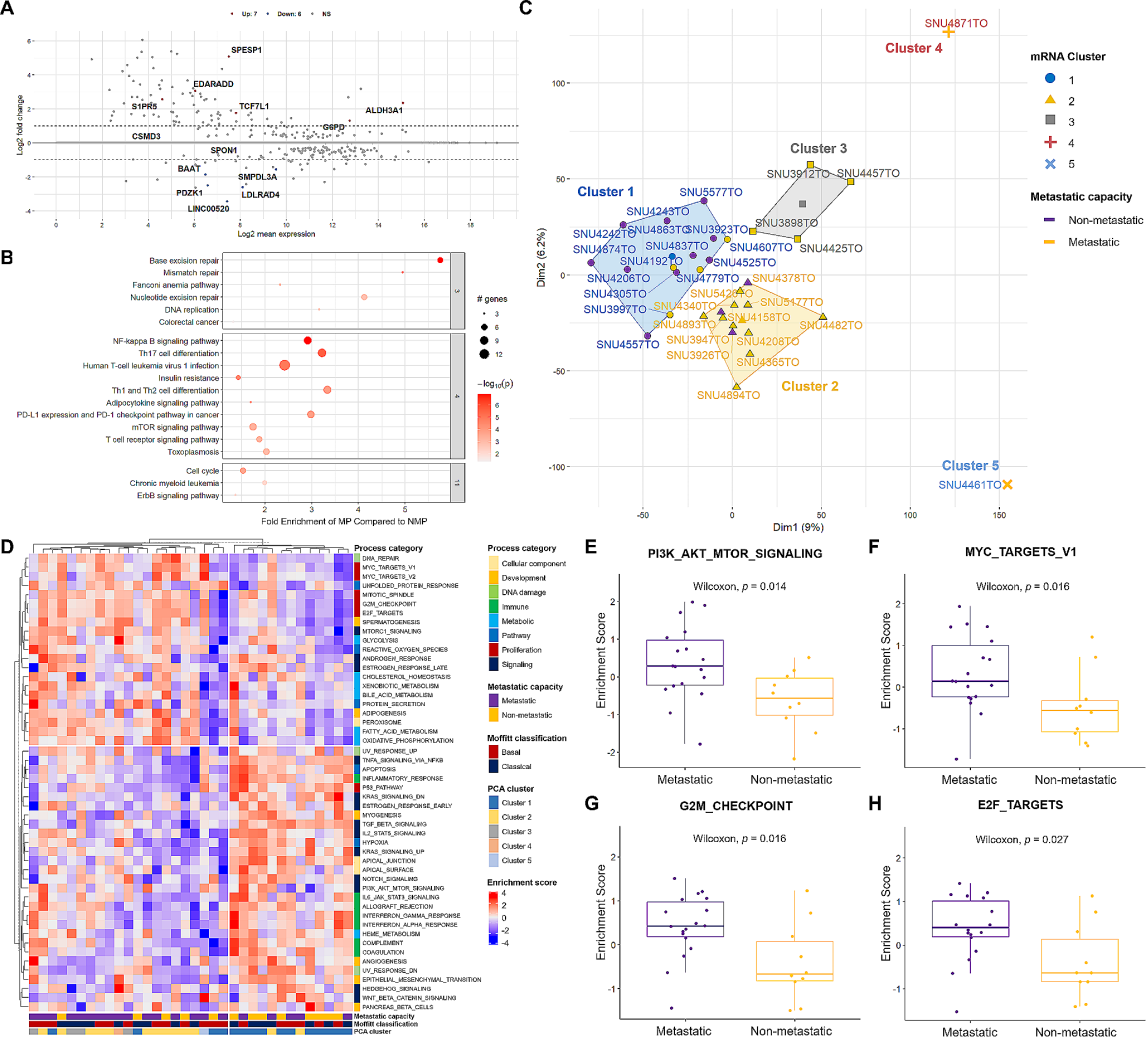
Transcriptomic Sub-Classification Revealed Distinct Pattern of PDACs with Metastatic Potency. See also Supplementary Table 4. **(A)** Differentially expressed gene (DEG) analysis identified seven up-regulated (Red) and six down-regulated (Blue) genes (*p* < 0.05). **(B)** Pathways analysis revealed multiple pathway enrichments of metastatic potential (MP) group compared to non-metastatic (NMP) potential group. The fold enrichment of each pathways was scaled on the x-axis. The size of dots represents the number of counted genes in the dysregulated gene database. The– log transformed *p* value was indicated by red color. Pathways were clustered according to the number of shared genes with representative pathways on the top. **(C)** Principle component analysis (PCA) using 27,656 expressional profiles identified three major mRNA clusters separating organoids with MP from NMP. Each mRNA cluster and metastatic potency were marked with representative colors. **(D)** The single sample gene set enrichment scores (ssGSEA) of each organoid using 50 hallmark gene set database were grouped with k-means (k = 2). Metastatic potency and mRNA cluster from PCA were specified on the x-axis. The process categories, metastatic capacity and mRNA cluster from PCA were designated with representative colors. Enrichment score was indicated with representative colors (Red; up-regulated, Blue; down-regulated). **E-H.** Wilcoxon rank sum test indicated that four HALLMARK pathways were enriched in organoid groups with metastatic potency

We also inspected transcriptomic features of PDAC organoids in order to identify distinct expressional patterns of PDACs with metastatic potency. We first analyzed differentially expressed genes in MP organoid groups compared to NMP organoids using DEseq2, which revealed seven up-regulated and six down-regulated genes (Fig. 3A). Among them, three genes (ALDH3A1, PDZK1, and LINC00520) were statistically related to patient survival in the TCGA PAAD cohort (Supplementary Fig. 5A-C). Higher mRNA expressions of ALDH3A1 and LINC00520 were associated with poorer overall survival, while a higher level of PDZK1 was indicative of better survival rates. We then used log2 fold change values and adjusted *p*-values for all genes (21,956 genes), which served as the direct input gene list for following pathway analysis. The pathfindR package utilizes both log2 fold change values and adjusted *p*-values to determine enrichment scores, considering both up-regulated and down-regulated genes within the specified pathway dataset (Supplementary Table 4A). Multiple clusters including DNA repair, immune-related, and cell cycle regulations were enriched in the MP group (Fig. 3B).

Since supervised approaches indicated that there were transcriptional variations between MP and NMP organoids, we re-validated this result using unsupervised methods. We first compared entire mRNA expression profiles (*n* = 27,656) of each organoid using principle component analysis (PCA), which separated MP from NMP organoids based on dimension 1 (9%) (Fig. 3C). Cluster #1 was mostly comprised of NMP organoids whereas cluster #2 and #3 consisted of MP organoids. We also estimated enriched pathway scores using 50 hallmark gene sets from molecular signature database [35]. We then determined the optimal number of clusters using elbow method, and grouped both organoids and process categories based on k-means (optimal *k* = 2) (Fig. 3D). The heatmap indicated that organoids with MP were likely to be separated in accordance with the metastasis status of each patient as well as the mRNA cluster from the PCA. In terms of the process category, a metastatic cluster displayed enriched proliferation and metabolic processes whereas non-metastatic cluster exhibited enriched immune and signaling processes. Other processes such as cellular component and development were not visibly grouped with metastatic potency.

We then performed Wilcoxon rank sum test in order to pinpoint which hallmark pathways were differently enriched, which revealed that four pathways (PI3K_AKT_MTOR, MYC_TARGETS_V1, G2M_CHECKPOINT and E2F_TARGETS) were significantly upregulated in the MP organoid group (Fig. 3E-H, Supplementary Table 4B). Connor et al. have reported that cell cycle progression increases with sequential inactivation of tumor suppressors, yet remains higher in metastases [12]. We further correlated genes previously reported to be involved in cell cycle progression with our pathway analysis. We identified gene lists for four target pathways: PI3K_AKT_MTOR_SIGNALING, MYC_TARGETS_V1, G2M_CHECKPOINT, and E2F_TARGETS. Of the 31 genes, 14 were associated with the G2M_CHECKPOINT pathways and 16 with E2F_TARGETS, corroborating that our findings align with prior research. PI3K_AKT_MTOR_SIGNALING and MYC_TARGETS_V1 shared only single gene with the previously reported genes, highlighting them as potential targetable pathways in metastatic PDAC (Supplementary Table 4C). Our findings suggest that the transcriptomic landscapes of PDAC organoids, derived from primary tumors, exhibit enriched cell cycle progression patterns akin to those anticipated in metastasized clones. This observation implies a molecular resemblance between primary PDACs exhibiting metastatic potential and the theoretical profiles of metastasized clones, as inferred from their shared expression patterns related to cell proliferation and metabolic pathways. Nevertheless, direct sequencing of metastasized clones was not part of this study. Therefore, the comparison is speculative and based on the premise that primary tumors with high metastatic potency may share key molecular features with actual metastatic lesions, warranting further investigation to directly compare these entities.

### Molecular features according to metastatic potency linearly correlate with responses to cytotoxic drugs

**Fig. 4:**
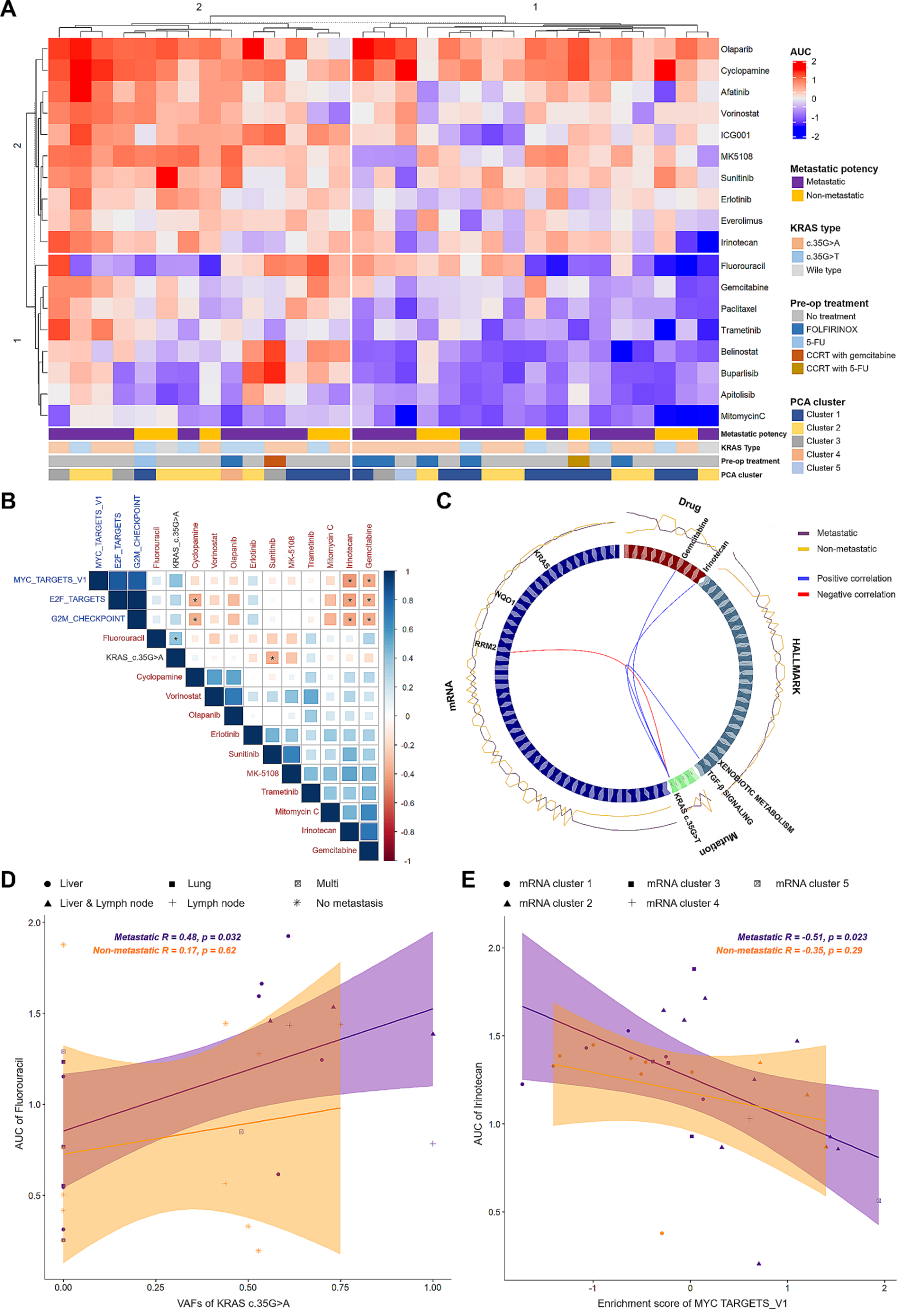
Integration of Metastatic Molecular Factors with Heterogeneous Drug Responses Reveals Linear Correlation to Cytotoxic Drugs. See also Supplementary Figs. 6, 7, Supplementary Table 5. **(A)** Drug responses of PDAC organoids exhibited heterogeneous distribution of 18 compounds. A total of 35 organoids were successfully screened in duplicate, generating > 1,200 measurements of drug interactions. The names of compounds are provided on the right. The organoids and drugs were k-means clustered based on the AUC values across the drug panel. Prospective molecular markers accounting for the metastatic potency were designated under the heatmap to visualize association with heterogeneous drug responses. AUC values ranging from − 2 (Blue; sensitive) to 2 (Red; resistant) and molecular markers were marked with representative colors. **(B)** The linear correlation of metastatic molecular factors with drug responses is indicated. The name of each factor is designated with different colors (Black; VAFs of *KRAS* c.35G > A, Blue; enrichment score of hallmarks pathways, Red; compounds). The Pearson correlation coefficient (R) with *p*-values between the molecular factors and AUC of 11 drugs are represented. (Blue; positive correlation, Red; negative correlation). Significance codes: ‘*’ *p* < 0.05. **(C)** Multi-omics analysis interlinked 4 different omics layers (Mutation, mRNA, Hallmark pathway, and drug response) centering on VAFs of *KRAS* c.35G > T. The correlation between two components is designated with the color of lines (Red, positive correlation; Blue, negative correlation). The correlation coefficient (R) cut-off was 0.77, and relative contribution of each component to organoid groups with MP and NMP is indicated around the circos plot. Each block is marked with representative colors. **(D)** The linear correlation between the VAFs of *KRAS* c.35G > A and the AUC of Fluorouracil is depicted. The Pearson correlation coefficient (*R*) with *p* value is calculated according to the metastatic potency and marked on the top of the correlation graph. Confidence intervals are calculated at a confidence level of 0.95 for the parameter and indicated by a shading along with the line. The specific metastasis sites were marked with different figures. **(E)** The linear correlation between the enrichment score of MYC TARGETS_V1 and the AUC of Irinotecan is depicted. The Pearson correlation coefficient (*R*) with *p* value is calculated according to the metastatic potency and marked on the top of the correlation graph. Confidence intervals are calculated at a confidence level of 0.95 for the parameter and indicated by a shading along with the line. The specific mRNA clusters were highlighted with different figures

We prepared an 18-drug screening library to measure heterogeneous drug responses of PDAC organoids. A total of 35 organoids were successfully screened in duplicate, generating > 1,200 measurements of drug interactions. The elbow method identified two major sub-groups among screened organoids and drugs. Organoid group 1 mostly displayed good response to drug group 1 consisting of Gemcitabine, Paclitaxel, and Mitomycin C. Organoid group 2 exhibited heterogeneous responses to these drugs. For drug group 2 consisting of Irinotecan, Cyclopamine and Sunitinib, organoid group 1 exhibited moderate resistance and organoid group 2 showed heterogeneous sensitivities (Fig. 4A). Pre-operative treatment can directly affect clonal composition of the original tumor, which might put strong bias in drug responses of organoids. We revealed that FOLFIRINOX treatment and subsequent response to Gemcitabine indeed implies a potential clustering or pattern within organoid group 1 (Fig. 4A). This observation suggests that prior exposure to FOLFIRINOX could influence the sensitivity of organoids to Gemcitabine as previous studies have indicated that using Gemcitabine as a second-line treatment for advanced pancreatic adenocarcinoma following FOLFIRINOX failure can offer clinical benefits in certain patients [36]. Prospective molecular markers accounting for metastatic potency were designated under the heatmap to visualize association with heterogeneous drug responses. As a first validation, metastatic potency and different *KRAS* mutation types were seldom correlated with drug groups, underlining further integration of genetic and transcriptomic markers with drug responses (Supplementary Table 5A). We also estimated gene-drug interactions including mutational status of 14 genes mostly mutated in pancreatic tumor (Fig. 2A) and responses to 18 drugs (Fig. 4A) using Wilcoxon rank sum test (Supplementary Fig. 6A, Supplementary Table 5B). This revealed two statistically significant gene-drug interactions (*p* < 0.05). This identified two statistically significant gene-drug interactions (*p* < 0.05). A mutation in KDM6A was associated with a poorer response to the MK5108 drug, and a BRCA1 mutation was linked to an improved response to Gemcitabine.

We then applied potential molecular markers in association with metastatic capability to estimate statistical correlation with drug responses. Three drugs (Irinotecan, Gemcitabine, and Cyclopamine) exhibited negative correlations with three transcriptomic factors (MYC_TARGETS_V1, E2F_TARGETS, and G2M_CHECKPOINT pathways). A genomic factor, the VAF of *KRAS* c.35G > A mutation, was directly correlated with fluorouracil and inversely associated with sunitinib (Fig. 4B). To interlink multi-omics layers, we integrated four representative blocks consisting of drug responses, VAF of driver mutations, enrichment score of hallmark pathways, and mRNA expression of drug-related genes [37] using DIABLO r package [38] (Fig. 4C). This integrative analysis identified clonal population of *KRAS* c.35G > T, a mutational factor, was the key linking the other three factors. The VAFs of *KRAS* c.35G > T was negatively correlated with mRNA expression of *RRM2* but positively associated with TGF-β signaling pathway as well as AUCs of Gemcitabine and Irinotecan (Fig. 4C and Supplementary Fig. 6B). Each correlation has been previously reported [39–42]. Nevertheless, the detailed mutational type of *KRAS* as the core regulation factor was unspecified. In terms of potential metastasis marker, VAFs of *KRAS* c.35G > T presented no statistical association with MP, yet mRNA expression of *RRM2* was distinctly higher in MP organoid groups, which implied the potential role of *RRM2* as a predictive marker of metastasis in primary PDACs. We also divided each factor with two components (Supplementary Fig. 6C) and estimated the contribution of each block in accordance with MP (Supplementary Fig. 7A, B). This revealed that component 1 separated MP from NMP groups and provided contributing blocks. In parallel with prior analysis, *KRAS* c.35G > A highly contributed to the separation of MP from NMP groups.

We further analyzed effects of MP markers with certain drugs by integrating other clinical factors as well as metastatic conditions. The Pearson correlation previously indicated that cellular fraction of the KRAS c.35G > A mutation was positively related to AUC of Fluorouracil (Fig. 4B). In our analysis, we observed no significant statistical correlation in the NMP organoid group (*R* = 0.17, *p* = 0.62) when examining the role of the KRAS c.35G > A mutation, suggesting a differential impact of this mutation across organoids with varying metastatic potencies. This observation raises the possibility that the cellular fraction of KRAS c.35G > A mutation might influence drug resistance patterns, notably to 5-FU, in a context-dependent manner, particularly within metastatic PDAC clones. However, this inference is based on correlational data and requires further experimental validation to understand the underlying mechanisms fully. Integrating specific metastasis sites revealed that majority of liver-metastasized samples (5 out of 7) were not plotted within the 95% confidence interval, implying that there could be stronger driving force for liver dissemination than KRAS c.35G > A mutation. Analogous pattern was observed with AUCs of irinotecan and enrichment score of MYC TARGETS_V1 (Fig. 4E). A reverse correlation between two factors (Fig. 4B) was not presented in NMP groups (*R* = -0.35, *p* = 0.29). Prior clustering by PCA (Fig. 3C) indicated that four MP organoids were grouped with NMP cluster (cluster 1). These organoids were plotted within 95% confidence interval of MP organoids. In contrast, NMP organoids grouped with MP clusters (clusters 2 and 3) in the PCA were plotted out of 95% interval, highlighting the specification of certain transcriptomic pathways for better correlation with drug responses. Our findings suggest the potential of utilizing specific molecular markers, such as KRAS mutations and RRM2 expression levels, as indicators for tailoring first-line therapeutic strategies. For instance, the variant allele frequencies of KRAS mutations, particularly the c.35G > A and c.35G > T variants, alongside RRM2 expression, could inform the selection of targeted drugs. These markers might guide the use of chemotherapeutic agents like Gemcitabine and 5-FU more effectively, depending on their association with metastatic potency in PDAC. Further research is needed to validate these markers as predictive tools for optimizing treatment regimens.

### Machine learning approach enables prediction of metastatic potency within primary PDAC using drug responses as well as molecular markers

**Fig. 5:**
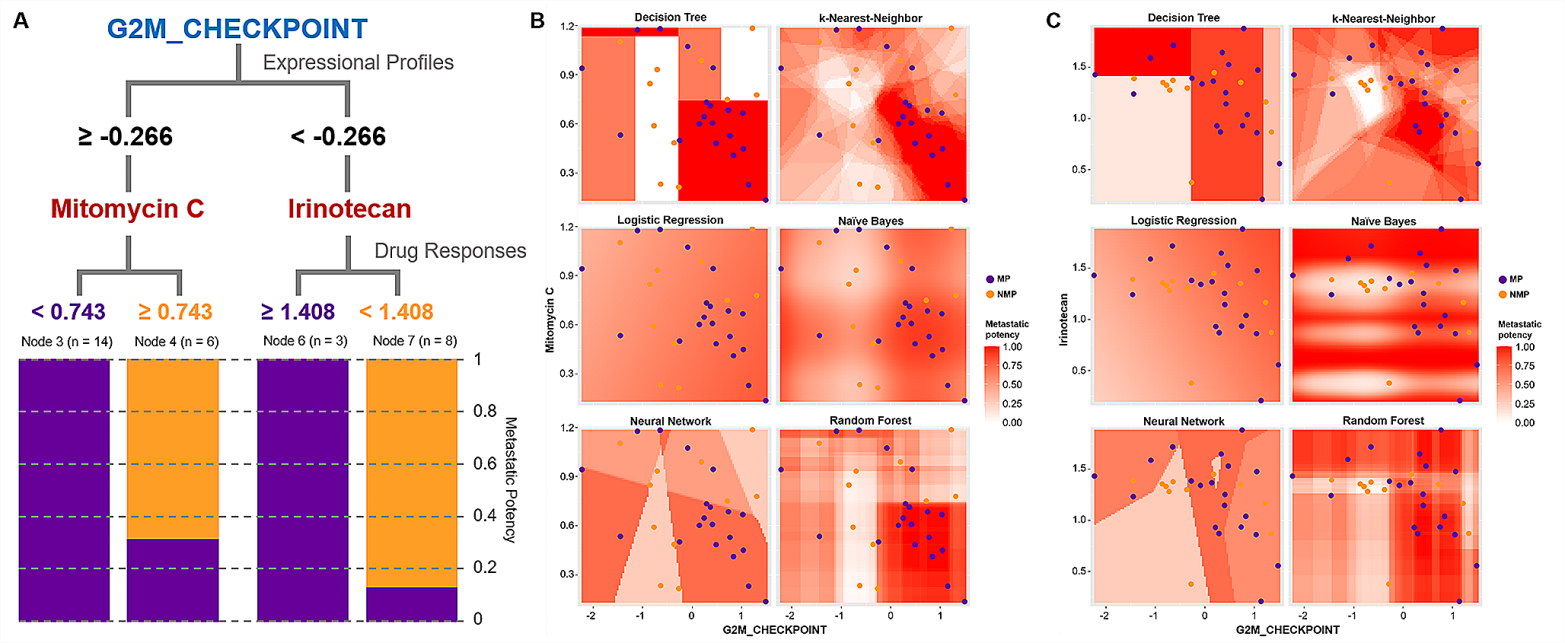
Machine Learning Approach Integrates Molecular Factors to Drug Responses to Predict Metastatic Potency. See also Supplementary Table 6. **(A)** Representative guides from the decision tree method integrate the expression profile with drug responses to estimate metastatic potency. The value of each factor separating MP groups from NMP groups is indicated along the branches of the tree. Each node is divided according to the metastatic potency with the representative colors (Purple; MP, Yellow; NMP). **(B)** Visualization of correlation between enrichment scores of G2M CHECKPOINT and AUCs of Mitomycin C using six different machine learning methods identified aggregating patterns of MP groups. The metastatic potency ranging from 0 to 1 was highlighted with red color. **(C)** Visualization of correlation between enrichment scores of G2M CHECKPOINT and AUCs of Irinotecan using six different machine learning methods identified aggregating patterns of MP groups. The metastatic potency ranging from 0 to 1 was highlighted with red color

Several studies have reported that patients who were diagnosed with primary PDAC are often progressed to metastatic stage even after the treatment [20]. This implies that responses of primary PDAC to certain drugs might be used to predict the presence of metastatic clones within the primary PDAC. Prior regression models provided direct comparisons between two variables, which enabled identification of potential molecular factors associated with metastatic potency (Fig. 4B-E). We additionally applied various machine learning methods including nearest neighbor, artificial network, random forest, and decision tree. We estimated the prediction power of each model with area under curve (AUC). Most models displayed comparable prediction power except for random forest (Supplementary Table 6). The decision tree branched out on the basis of previously identified expressional factor, enrichment score of the G2M_CHECKPOINT. Our analysis revealed that up-regulation of the G2M_CHECKPOINT (NES ≥ -0.266) correlates with a higher metastatic propensity in organoids that are sensitive to DNA damage-induced stress, as indicated by their response profiles to cytotoxic agents. This suggests that the activation of cell cycle checkpoints might serve as a biomarker for aggressive disease behavior and potential resistance to standard therapies. While Mitomycin C itself is not a mainstay in PDAC treatment, this finding underscores the importance of targeting cell cycle and DNA repair pathways in developing novel therapeutic strategies for PDAC with high metastatic potential (Fig. 5A). The overall correlation between high proliferation of tumor cells due to activated G2M checkpoint and increased metastatic potency was demonstrated in our transcriptomic analysis (Fig. 3G), consistent with a previous study [12], which linked good responses of Mitomycin C to vulnerabilities of potential metastasis. This clinically implies that PDAC patients who exhibit good sensitivities to pre-operative Mitomycin C should be cautiously watched out for potential dissemination of tumors. On the contrary, when G2M_CHECKPOINT was comparatively down-regulated (NES < -0.266), the organoid group which displayed insensitivities to Irinotecan (AUC ≥ 1.408) had higher metastatic potency (Fig. 5A). A prior study has reported that resistance to Irinotecan is induced from deregulated acetylation of H4K16 [43] which maintains multiple gene expressions involved in cell motility [44]. This suggestes the potential role of epigenetic regulation in irinotecan-resistant tumor cells. We visualized the predicted metastatic potency using six different machining learning models setting G2M_CHECKPOINT as a common denominator (Fig. 5B, C), which demonstrated that regression approach was unable to precisely determine metastatic groups even when each factor was identified by the linear regression. This revalidated the necessity of using several statistical models to integrate multiple molecular factors and phenotypical feature to draw generalizable conclusion. To validate our prediction model of metastatic potency using drug responses and molecular markers, we conducted in vitro invasion assays as an indicative of metastatic capability. Utilizing the prediction model based on responses to Irinotecan and Mitomycin C, as well as enrichment scores for G2M_CHECKPOINT, we analyzed three metastatic potential (MP) organoids (SNU-4158-TO, SNU-4461-TO, and SNU-4871-TO) and two non-metastatic potential (NMP) organoids (SNU-4354-TO and SNU-5577) for invasion capabilities. Our results demonstrated that two of the MP (metastatic potential) organoids showed significant invasion into the matrix, whereas none of the NMP (non-metastatic potential) organoids exhibited invasion (Supplementary Fig. 8A, B and C). Cell proliferation was measured concurrently with the 3D spheroid cell invasion assay to differentiate invasion ability from growth rate. After 96 h, the growth rates among the organoids showed little variation, indicating that the differences in invasion capability were not due to the proliferation rate (Supplementary Fig. 8D). It should be noted that while in vitro invasion assays can provide insights into the potential for metastasis, they do not fully model the entire metastatic process.

Additionally, we conducted immunocytochemical analyses to evaluate the expression of markers associated with metastatic potential, utilizing anti-Vimentin (red) and anti-E-cadherin (green) antibodies. The results demonstrated that SNU-4458-TO and SNU-4871-TO, characterized by pronounced invasive traits in the invasion assays, manifested elevated Vimentin expression across the organoid structure (Supplementary Fig. 9A, C). Conversely, SNU-4354-TO exhibited greater E-cadherin expression relative to Vimentin (Supplementary Fig. 9B, C). SNU-5577-TO showed comparable levels of Vimentin and E-cadherin expression (Supplementary Fig. 9B, C).

## Discussion

Pancreatic ductal adenocarcinoma (PDAC) is a highly aggressive disease accompanied by a frequent metastatic burden at the time of diagnosis [45]. Early dissemination of PDAC mainly causes dismal prognosis, largely limiting available treatment options [46]. Metastatic spread of PDAC can be initiated during early phase of tumor formation [18, 47], which implies that borderline resectable PDAC comprising metastatic clones might exhibit distinguishable molecular patterns.

In this study, we established 36 organoids from primary tumor tissues of PDAC patients with varying metastatic statuses to capture metastatic potency (MP) of primary PDAC. All organoids introduced in this study will be bio-banked and distributed worldwide. Genetic analysis of these models demonstrated that several mutational features were shared between MP and NMP organoids. For instance, the mutational frequencies of TP53 in MP and NMP organoids were 55% and 62%, respectively (Fig. 2A). Additionally, the overall mutational signatures were similar between MP and NMP organoids (Fig. 2B-E). We focused on the accumulation of the KRAS c.35G > A mutation, indicated by higher variant allele frequencies (VAFs) in MP organoids compared to NMP organoids. Transcriptomic analysis of our models also revealed upregulation of cell proliferation and metabolic pathways in MP organoids through linear correlation. However, it should be noted that these pathway analyses were solely derived from RNA-sequencing analyses and further correlation studies, acknowledging the absence of direct in vitro experimental validation for increased cell proliferation or metabolic signaling in MP versus NMP organoids. Our pharmacological analysis demonstrated that several MP-specific molecular factors were linearly correlated with cytotoxic drugs. For instance, the variant allele frequencies (VAFs) of the KRAS c.35G > A mutation were positively correlated with 5-FU sensitivity and negatively correlated with Sunitinib sensitivity. Additionally, enrichment scores of the E2F_TARGETS and G2M_CHECKPOINT pathways were inversely correlated with Cyclopamine, Irinotecan, and Gemcitabine sensitivity. In a few cases, Pearson correlations were statistically significant only within MP organoid groups, demonstrating their values as actionable therapeutic targets of metastasized PDAC. Our multi-omics analysis also identified the KRAS c.35G > T mutation as a key factor linking the drug responses to Gemcitabine and Irinotecan, as well as the mRNA expression of RRM2. Notably, it has been demonstrated that upregulation of RRM2 leads to Gemcitabine chemo-resistance in pancreatic cancer (PC) cells and human PC xenografts in mice [48]. Additionally, the expression level of RRM2 was inversely correlated with overall survival (OS) in Gemcitabine-treated PC patients in a clinical study [49]. Our multiblock analysis linked the previously reported correlation of mRNA expression of RRM2 to Gemcitabine resistance with the KRAS c.35G > T mutation as a key connecting factor. In addition, our machine learning models enabled to estimate metastatic potency of primary PDAC using heterogeneous drug responses of Mitomycin C and Irinotecan.

Few NMP organoids displayed enriched EMT, TGF-beta signaling, and KRAS signaling up pathways, which seemingly contradicts their associations with invasive tumor features typically observed in more aggressive, metastatic cancers. One possible explanation for the enrichment of EMT and TGF-beta signaling pathways in NMP organoids could be the presence of a cellular context in which these pathways are activated but do not lead to the expected invasive behavior, possibly due to the activation of counter-regulatory mechanisms or the absence of additional co-factors required for metastasis initiation. Regarding the upregulation of KRAS signaling in NMP organoids, it is well-established that KRAS mutations are a hallmark of PDAC, promoting tumor growth and survival. The differential enrichment of KRAS signaling between NMP and metastatic potency (MP) organoids may reflect that while KRAS activation is necessary for tumorigenesis, its role in metastasis could be modulated by other genetic or epigenetic factors present in the tumor microenvironment. Further investigation is required to understand the implications of these findings fully, including detailed functional studies to dissect the roles of EMT, TGF-beta, and KRAS signaling in the context of PDAC metastasis.

With only 36 organoids from PDAC patients, our study may not fully capture the heterogeneity of PDAC, especially considering the diverse metastatic statuses and genetic backgrounds of the patients. This limitation is critical in the context of validating the effectiveness of the prediction model, as a larger and more varied clinical cohort could provide a more accurate assessment of the model’s predictive power across different PDAC subtypes. The lack of internal and external validation further compounds this issue, suggesting that future research should aim to include a larger sample size that encompasses a broader spectrum of PDAC cases.

Taken together, our approach provides a prospective pancreatic tumor organoid biobank and large-scale omics to understand mechanisms by which PDAC cells gain their metastatic potency. By capturing genetic and transcriptomic features of PDACs with live sets of organoids, which retained histopathological and molecular profiles of human PDAC, our data revealed that PDACs even diagnosed with resectable or borderline stage harbored distinct molecular profiles of metastatic potency, mediating prevalent heterogeneous drug responses. Our data also suggest that sole molecule-based prediction of PDAC dissemination has fundamental drawback. As a potential solution, our data emphasize the importance of connecting molecular profiles to responses of cytotoxic drugs to defy potential metastasis.

## Materials and methods

### Sample collection and preparation

We collected a total of 36 specimens of human pancreatic ductal adenocarcinoma (PDAC) from 36 different patients who underwent surgical resection or endoscopic ultrasound-guided fine-needle aspiration (EUS-FNA) at Seoul National University Hospital (Seoul, Korea).

All EUS-FNA procedures were conducted by a skilled echoendoscopist, who annually performs over 100 EUS-FNA procedures. Utilizing a linear EUS scope (GF-UCT260; Olympus Medical Systems, Tokyo, Japan) equipped with either a 19- or 22-gauge needle (EZ Shot 3 Plus; Olympus Medical Systems), the choice of needle size was at the endoscopist’s discretion. To obtain tissue samples for preliminary diagnosis, the needle was moved back and forth 15 times during 2 or 3 passes, employing a 20 mL suction syringe to ensure the collection of sufficient tissue. These samples were then forwarded to the department of pathology for the preparation of formalin-fixed paraffin-embedded tissue blocks and subsequent routine diagnostic evaluation. For the specific purpose of creating organoids, only one additional pass of the needle was executed. Samples collected for research were immediately placed in Opti-MEM supplemented with 1% Penicillin/Streptomycin (GIBCO, CA, USA, Cat#15140-122) and transported to the department of biomedical sciences (Korean Cell Line Bank at Seoul National University College of Medicine) to facilitate the development of pancreatic cancer organoids. The aspirated tissue was directly subjected to enzyme digestion.

For the surgically resected tumor, tissues were histologically diagnosed by a pathologist as adenocarcinoma. The surgically resected tumor tissue was submerged in Opti-MEM supplemented with 1% Penicillin/Streptomycin (GIBCO) and transferred in an ice box directly from the operating room to the laboratory. The volume of tissue fragments obtained from surgical resections ranged from 0.2 cm³ to 0.5 cm³ and were finely minced with surgical scissors before undergoing enzyme digestion. The success rates for establishing organoids from surgical resection and EUS-FNA were 37% and 60%, respectively.

Since the size of tumor obtained from both surgical resection and EUS-FNA was highly limited, the comparison between the original tumor tissue and organoids was performed in eight representative pairs. Detailed information about tumor sizes and locations is summarized in Table 1. A living biobank of PDAC organoids is cryopreserved at Korean Cell Line Bank (KCLB, http://cellbank.snu.ac.kr, http://organoid.snu.ac.kr) and will be distributed worldwide.

### Tumor isolation and initial cultivation of organoid

Tumors were chopped and incubated with collagenase II (1.5 mg/mL) (GIBCO, Cat# 17101-015), hyaluronidase (20 µg/mL) (Sigma-Aldrich, MO, USA, Cat# H3506) and Ly27632 (10 µ_M_) (Selleckchem, TX, USA, Cat# S1049) for 30–60 min depending on the size of tumor piece at 37℃ on a shaker. Basal culture medium (advanced Dulbecco’s Modified Eagle Medium/Ham’s F-12 supplemented with 1% penicillin and streptomycin, 10 mM HEPES and Glutamax) with 10% FCS was added and the mixture was passed through a 100 µM cell strainer to remove debris or clumps. Then, red blood cell (RBC) lysis buffer (2–5 mL) (Sigma-Aldrich, cat# R7757) was added for 1–5 min to remove RBCs. Cells were spun down at 1,000 rpm for 3 min, and re-suspended in BME (GIBCO, Cat# A14132-02) for seeding in a T-25 flask (Corning, NY, USA, Cat# 353,108). Approximately 5–7 BME domes (each consisting of BME (50 µL) containing 20,000 cells/mL) were seeded in a single T-25 flask. The flask was incubated at 37℃ for 10 min. Once BME had solidified, 5 mL of culture medium was added, and cells were incubated in a 37℃ and 5% CO_2_ culture incubator. The culture medium consisted of 40% W/V basal culture medium, 50% W/V L-WRN conditioned medium, 1 x B27 (GIBCO, Cat# 17504-044), human EGF (50 ng/mL) (GIBCO, Cat# PHG0313), human FGF-10 (10 ng/mL) (Peprotech, Cat# 100 − 26), nicotinamide (10 m_M_) (Sigma-Aldrich, Cat# 72,340), N-acetylcysteine (1.25 m_M_) (Sigma-Aldrich, Cat# A7250), A83-01 (500 n_M_) (Sigma-Aldrich, Cat# SML0788), and primocin (100 µg/mL) (GIBCO, Cat# ant-pm-1).

### Pancreatic cancer organoid cultures and passaging

The culture medium was refreshed every two to five days depending on growth rate. Organoids were photographed at initial passages (p1-p3). For passaging, the BME dome was mechanically pipetted using TrypLE Express solution (GIBCO, Cat# 12604-021) and organoids were collected in a 15 mL conical tube. The BME dome was mechanically dissociated with intense pipetting, and the tube containing the organoids and BME mixture was incubated at 37 °C for approximately 5–10 min. The organoids were centrifuged at 1,000 rpm for 3 min, and the supernatant was aspirated. Once BME was removed, the cell pellet was resuspended with fresh BME, seeded in a T-25 flask and the flask was incubated at 37℃ for 10 min. Once BME was solidified, the culture medium (5 mL) was added to the flask to overlay the BME dome and cells were incubated in a 37℃ and 5% CO_2_ culture incubator.

### H&E staining and immunocytochemistry

Tumor tissues were fixed in 10% neutral buffered formalin and embedded in paraffin. Then, tissues were sectioned at 4 μm thickness. For organoids, BME dome was mechanically scraped with a pipet tip. Cold PBS (10 mL) was added to collect dissociated BME domes and transferred to a 15 mL conical tube. After 15 s centrifugation at 100 rpm, the supernatant was aspirated. This procedure was repeated until the BME gel was visibly removed. Care was taken not to destroy the original structure of the organoids. Collected organoids were embedded in 2% agarose gel (INTRON Biotechnology, Seongnam, Korea). Solidified agarose gel was fixed in 10% formalin for 30 min at room temperature and sectioned at 4 μm thickness. Sections were subjected to H&E as well as immunohistochemical staining. For immunocytochemistry, organoids in their passages 1–3 were used. BME dome was mechanically scraped with a pipet tip. Cold PBS (10mL) was added to collect dissociated BME domes and transferred to a 15 mL conical tube. After 100 rpm, 15 s centrifugation, the supernatant was aspirated. This procedure was repeated until the BME gel was visibly removed. Care was taken not to destroy the original structure of the organoids. Then, organoids were fixed and permeabilized with BD Cytofix/Cytoperm (BD Science, CA, USA). After cells were washed with washing solution (BD Science), DPBS containing 2% FBS (GE Healthcare Life Sciences, Buckinghamshire, UK) was applied for an hour. After organoids were washed with cold DPBS, E-cadherin (Abcam, Cambridge, UK) (1:1000), Vimentin (Abcam) (1:500) and KI-67 (Abcam) (1:500) diluted in 0.05% of PBS.T was applied for 1.5 h at room temperature. Thereafter, cells were washed with 0.05% of PBS.T, and Alexa 488 secondary antibodies (Thermo Fisher Scientific) (1:500) and Alexa 568 secondary antibodies (Thermo Fisher Scientific) (1:500) diluted in 0.05% of PBS.T were applied for an hour at room temperature. DAPI (1:100) and rhodamine-conjugated phalloidin (Sigma-Aldrich, 1:10) were diluted in distilled water and applied for 30 min at room temperature. Cells were washed with DPBS three times and placed under a confocal microscope. LSM800 Confocal Laser Scanning Microscope and ZEN software (Carl Zeiss, Oberkochen, Germany) were used to analyze images. Digital resolution, scan speed, and the number of pictures averaged were set to 1024 × 1024, 40 s per one channel, and 8 pictures, respectively. Diverse magnifications were used in accordance with growth patterns and sizes of cells. The intensity of each channel was fixed for comparing target protein expression between samples. The ImageJ plugin “RGB-Measure” was utilized to quantify the intensity of each color channel. The overall intensity of E-cadherin was normalized by dividing the intensity of E-cadherin expression (green channel) by the intensity of DAPI expression (blue channel).

### 3D organoid cell invasion assay and proliferation assay

The organoid invasion assay was performed using the Cultrex 96-Well 3D BME Cell Invasion Assay kit (Sigma-Aldrich). A BME dome containing 20,000 cells/mL was mechanically pipetted with 1 mL of organoid culture medium. After centrifugation for 15 s at 100 rpm, the supernatant was gently aspirated. This procedure was repeated until the BME gel was visibly removed, taking care not to destroy the original structure of the organoids. The organoids were resuspended in 300 µL of Invasion Matrix included in the Cultrex 96-Well 3D BME Cell Invasion Assay kit. The 3D Culture Qualified 96-Well Plate was placed on ice for 15 min to cool the wells. Working on ice, 50 µL of the Invasion Matrix containing organoids was added to each well of the 3D Culture Qualified 96-Well Plate. The plate was centrifuged at 100×g at 4 °C for 1 min in a swinging bucket rotor to eliminate bubbles and position the organoids within the Invasion Matrix towards the middle of the well. The plate was then transferred to a tissue culture incubator set at 37 °C for 1 h to promote gel formation of the Invasion Matrix. After 1 h, 200 µL of pre-warmed (37 °C) organoid culture medium containing 10% FBS as the chemoattractant was added. The organoid in each well was photographed 24 and 96 h after initial seeding under a phase contrast microscope (Thermo Fisher Scientific). Images were analyzed using ImageJ version 1.54 g (http://rsb.info.nih.gov/ij/). Organoid proliferation was assessed concurrently with the 3D spheroid cell invasion assay to distinguish invasion ability from growth rate. Organoids were mechanically and enzymatically dissociated into single cells by incubating in TrypLE (Gibco) for 5 to 10 min. Suspension (5 µL/well) was dispensed in clear-bottomed, white-walled 96-well plates (#3903, Corning) using an automated repeat pipet and overlaid with 200 µL of a 1:1 mixture of culture medium and RGF basement membrane matrix (Gibco, A14132-02). Plates are incubated at 37 °C with 5% CO2 for 15 min to solidify the gel before addition of 20 µL of pre-warmed culture medium to each well. Proliferation was evaluated using the CellTiter-Glo® assay (Promega) in accordance with the manufacturer’s instructions after an incubation period of 24–96 h at 37 °C and 5% CO2. The entire procedure was independently duplicated.

### Whole exome sequencing analysis

The passages of organoids used for whole exome sequencing analysis were p2– p5. Total DNA was isolated from the organoids pellet using QIAamp DNA Mini Kit (Qiagen, Hilden, Germany) according to the manufacturer’s protocol. The Tissue DNA was extracted using QIAamp Fast DNA Tissue Kit (Qiagen, Hilden, Germany) according to the manufacturer’s protocol. We utilized the 2100 Bioanalyzer (Agilent, Part# G2939BA) to assess the degree of fragmentation of the genomic DNA sample by measuring the distribution of signal intensities across various sizes. SNU-2543-TO was excluded from the WES analysis due to low DNA integrity. Whole-exome capture was performed on all samples with the SureSelect Human All Exon V5 Kit (Agilent Technologies, Tokyo, Japan). The captured targets were subjected to sequencing using HiSeq 2500 (Illumina, San Diego, CA, USA) with the pair-end 100 bp read option for organoid samples and 200 bp read option for tissue materials. The sequence data were processed through an in-house pipeline. Briefly, paired-end sequences were aligned to the human reference genome (UCSC assembly hg19 - original GRCh37 from NCBI, 2009) using the mapping program BWA (version 0.7.12) [50], and generated a mapping result file in BAM format using BWA-MEM. PCR duplicates were removed using MarkDuplicates.jar included in Picard tools (v. 1.130, https://broadinstitute.github.io/picard/). The Genome Analysis Toolkit (GATK, v.3.4.0) [51] was used to performed base quality score recalibration (BQSR) and local realignment around indels. Somatic mutations were identified by providing sequence alignment data of tumor and normal to the MuTect (involved in GATK v3.8.0) with default parameters using tumor-normal mode taking both SNVs and short indels into account. We used hg19 as a reference genome build. Those variants are annotated by SnpEff v4.1 g, to vcf file format, annotating with dbSNP for the version of 142 and SNPs from the 1000 genome project. Then, SnpEff was applied to annotate additional databases, including ESP6500, ClinVar, dbNSFP 2.9. Mutational signatures were calculated using the MutationalPatterns R package v.3.4.0 [52] to detect distinct footprints in genomic context of somatic SNVs and evaluate mutational mechanisms. The relative contribution of mutational patterns designated multiple mutational signatures per each sample. The algorithm estimating the relative contribution of substitution patterns was to fit 96 types of substitution into previously constructed mutational signature. Therefore, a single substitution can be assigned to multiple signature, which is likely to be interpreted to other signatures.

### Analysis of RNA sequencing

Total RNA was isolated from cell lysate using TRIzol (Qiagen, Hilden, Germany) and Qiagen RNeasy Kit (Qiagen, Hilden, Germany). Paired-end sequencing reads from cDNA libraries (101 bp) were generated with an Illumina NovaSeq6000 instrument and the sequence quality was verified with FastQC v.0.11.7 (https://www.bioinformatics.babraham.ac.uk/projects/fastqc/). For data preprocessing, low quality bases and adapter sequences in reads were trimmed using Trimmomatic v 0.38 [53]. The trimmed reads were aligned to the human genome (UCSC hg19) using HISAT v2.1.0, a splice-aware aligner [54]. Then, transcripts including novel splice variants were assembled with StringTie v1.3.4d [55]. The abundance of these transcripts in each sample was calculated as read-counts or TPM (Transcript per Million mapped reads) values. For each sample, the transcript expressions were normalized by dividing read counts into lengths of the mapped genes. For Moffitt classification, we extracted and normalized the representative 25 Basal-like and 25 Classical signature genes to a Z-score [56]. Total score was calculated by subtracting the total Z-score of “Classical” genes from the total Z-score of “Basal-like” genes; PDOs with total score ≥ 0 were classified as “Basal-like” subtype, and PDOs with total score < 0 as “Classical” subtype. The comparison between samples with metastatic potential (MP) and non-metastatic potential (NMP) was performed using the DESeq2 [57] package to compute log2 fold changes and corresponding *p*-values for each gene. These genes were then utilized to generate an MA-plot and conduct pathway analysis with the pathfindR package [58]. For principal component analysis (PCA), the FactoMineR and factoextra packages were employed. Utilizing the raw read counts of 27,685 transcripts, a single-sample enrichment analysis of cell signaling pathways was conducted using GSEA (v4.1.0) and the Molecular Signatures Database (MSigDB) hallmark gene sets library [57]. The phenotype label was assigned as either metastatic potential or non-metastatic potential for the normalized enrichment score (NES) of a single sample. Independent NES for the sample was calculated using default settings with 1000 permutations for the phenotype. Results were annotated using the NCBI Gene ID and MsigDB v7.4 chip platform. Heatmaps of NES values were plotted using the ComplexHeatmap (v2.2.0) R package, with color variance mapped between the minimum and maximum values.

### 3D organoids seeding/drug treatment procedure

All drug screens were performed two times. We have mainly referred to National Cancer Institute (NCI) drug lists for pancreatic cancers (https://www.cancer.gov/about-cancer/treatment/drugs/pancreatic) for constructing the drug screen library. We also included fluorouracil and gemcitabine in the screening library as most patients received FOLFIRINOX (fluorouracil, leucovorin, irinotecan, and oxaliplatin) and gemcitabine treatment. Organoids were mechanically and enzymatically dissociated into single cells by incubating in TrypLE (Gibco) for 5 to 10 min. Suspension (5 µl/well) was dispensed in clear-bottomed, white-walled 96-well plates (#3903, Corning) using an automated repeat pipet and overlaid with 200 µl of a 1:1 mixture of culture medium and RGF basement membrane matrix (Gibco, A14132-02). Plates are incubated at 37 °C with 5% CO2 for 15 min to solidify the gel before addition of 20 µl of pre-warmed culture medium to each well. 96 h after seeding, 20 µl of drug containing solution is added to each well. For the control well, the mixture of culture medium and drug-solvent solution is added. The list and detailed information of drugs are listed below.

**Table:** 

Drugs	Company	Cat No.	Stock Quantities (mg)	Solvent	Max concentration (µ_M_)
Everolimus	Selleckchem	S1120	10	DMSO	100
Paclitaxel	Selleckchem	S1150	50	DMSO	50
5-FU	Sigma-Aldrich	F6627	1000	DMSO	50,000
Gemcitabine Hydrochloride	Selleckchem	S1149	25	DMSO	100
Irinotecan	Selleckchem	S2217	25	DMSO	100
Mitomycin C	Selleckchem	S8146	10	DMSO	100
Sunitinib Malate	Selleckchem	S1042	50	DMSO	100
Erlotinib Hydrochloride	Selleckchem	S1023	100	DMSO	100
MK-5108	Selleckchem	S2770	10	DMSO	100
Buparlisib (BKM120)	Selleckchem	S2247	10	DMSO	100
Apitolisib (GDC0980)	Selleckchem	S2696	10	DMSO	50
Vorinostat (SAHA)	Selleckchem	S1047	200	DMSO	50
Belinostat (PXD101)	Selleckchem	S1085	50	DMSO	100
Trametinib (GSK1120212)	Selleckchem	S2673	10	DMSO	50
Afatinib	Selleckchem	S1011	10	DMSO	50
Cyclopamine	Selleckchem	S1146	10	EtOH	50
Foscenvivint (ICG-001)	Selleckchem	S2662	25	DMSO	100
Olaparib	Selleckchem	S1060	25	DMSO	50

### ATP detection assay and statistical analysis of drug responses

After 72 h of drug treatment, 10 µl of 3D Reagent (Promega #G968B) is added to each well followed by 5 min of vigorous shaking. After 30 min incubation at room temperature and an additional minute of shaking, luminescence is measured with a Luminoskan Ascent (Thermo Scientific) over 1000 ms of integration time. The ATP detection level from vehicle-treated samples was used for normalization. The responses of the other drugs were calculated as AUC values and visualized with k-means clustered heatmap from ComplexHeatmap package (v. 2.13.0) from R program version 4.2.0 (R Foundation for Statistical Computing, Vienna, Austria). Optimal number of clusters for derivatives and drugs was calculated with factoextra package (v. 1.0.7) with elbow method from R program (R Foundation for Statistical Computing). For hierarchical cluster analysis on a set of dissimilarities, each object was assigned to its own cluster, which an algorithm proceeds through iteratively. Two of the most similar clusters are joined at each stage until there is a single cluster. Euclidian distances between clusters are recomputed at each stage by the Lance–Williams dissimilarity update formula according to the single linkage method.

### Multi-omics integration analysis

The drug response data is integrated with variant allele frequencies (VAFs) of pathogenic mutations, mRNA expressions, and pathway enrichment scores (NES) using mixOmics R Bioconductor package [38] with built-in analyzing and visualization functions. We have limited the construction matrix with 4 parameters: VAFs (6) x mRNA (377) x NES (50) x drug responses (18) estimating more than two million multi-omics combinations. We focused on the expressional profiles of drug-target genes referring to Drug–Gene Interaction Database (DGIdb 4.0) [37] and DrugBank database [59]. We first built a pseudo design matrix utilizing multiblock Partial Least Squares (PLS)- Discriminant Analysis (DA) to identify correlated variables across four different input datasets. Each numeric values from VAFs, mRNA, NES, and drug responses (latent components or linear combinations of variables) were constructed such that the sum of covariances between all pairs of datasets is maximized. Then, we estimated the relationship structure between the various inputted data, where each value (between 0 and 1) represents the strength of the relationship among three given data-frames. All pairwise covariances were weighted as indicated by the design matrix. The response variable is transformed into a dummy variable internally within the function. The regression sGCCA framework from the RGCCA package is utilized to deflate each of the datasets. The transformed variables were combined for further discrimination and integration.

### Construction of prediction model

All estimation and visualization were performed through R packages. Six different machine learning models including Decision tree [60], k-Nearest-Neighbor, Logistic regression, Naïve bayes, Neural network, and Random forest were applied to find best performance. Comparison and visualization was done by modelr package. The estimation of accuracy and confusion matrix is done by yardstick within tidymodels package to quantify how well model fits to a data set. Detailed information about input parameters are summarized in the code availability section. The genomic features to train the machine learning models are selected using pathway enrichment score (NES) and variant allele frequencies of mutations. We also included the responses of 18 drugs in the unit of AUC for training models.

## Electronic supplementary material

Below is the link to the electronic supplementary material.

Supplementary Material 1

Supplementary Material 2

Supplementary Material 3

Supplementary Material 4

Supplementary Material 5

Supplementary Material 6

Supplementary Material 7

## Data Availability

Organoids generated in this study have been deposited to the Korean Cell Line Bank (KCLB, http://cellbank.snu.ac.kr, http://organoid.snu.ac.kr) biobank and are governed by Ja-Lok Ku.

## Code availability

Raw FASTQ files in accordance with the MINSEQE standards will be deposited in a Sequence Read Archive (SRA) and will be freely available. Few processed sequencing data will be available by request and governed by the Lead Contact. Computational pipelines as well as sequencing data including whole exome sequencing and RNA-sequencing in this study are available at the public repository: https://github.com/mario2437/Pancreatic_Tumor_Organoid_1st.
