# Supplementary material for: Establishment, characterization, and biobanking of 36 pancreatic cancer organoids: prediction of metastasis in resectable pancreatic cancer
Source: Cell Oncol (Dordr). 2024 Apr 15;47(5):1627–47. doi: 10.1007/s13402-024-00939-5 (PMC11467084; doi:10.1007/s13402-024-00939-5)
Supplement: Supplementary file 1 — Supplementary Material 1 [file 13402_2024_939_MOESM1_ESM.docx]

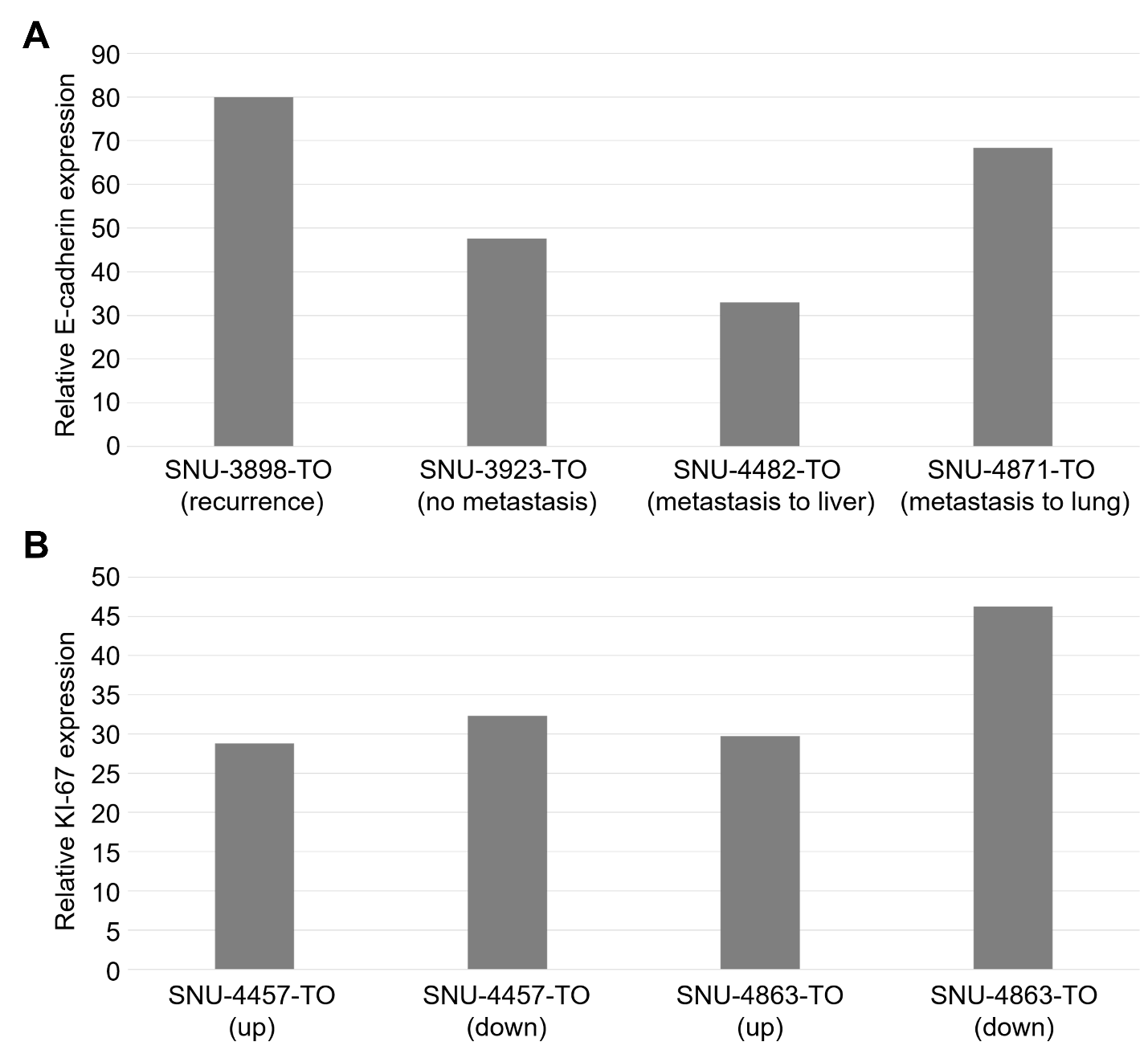


**Supplementary Fig. 1A.** Histopathological comparison between PDAC organoids derived from the surgical resection and the original tissue. **1B.** Histopathological comparison between PDAC organoids derived from the fine needle aspiration biopsy and the original tissues. The size of scale bar is indicated in each slides.


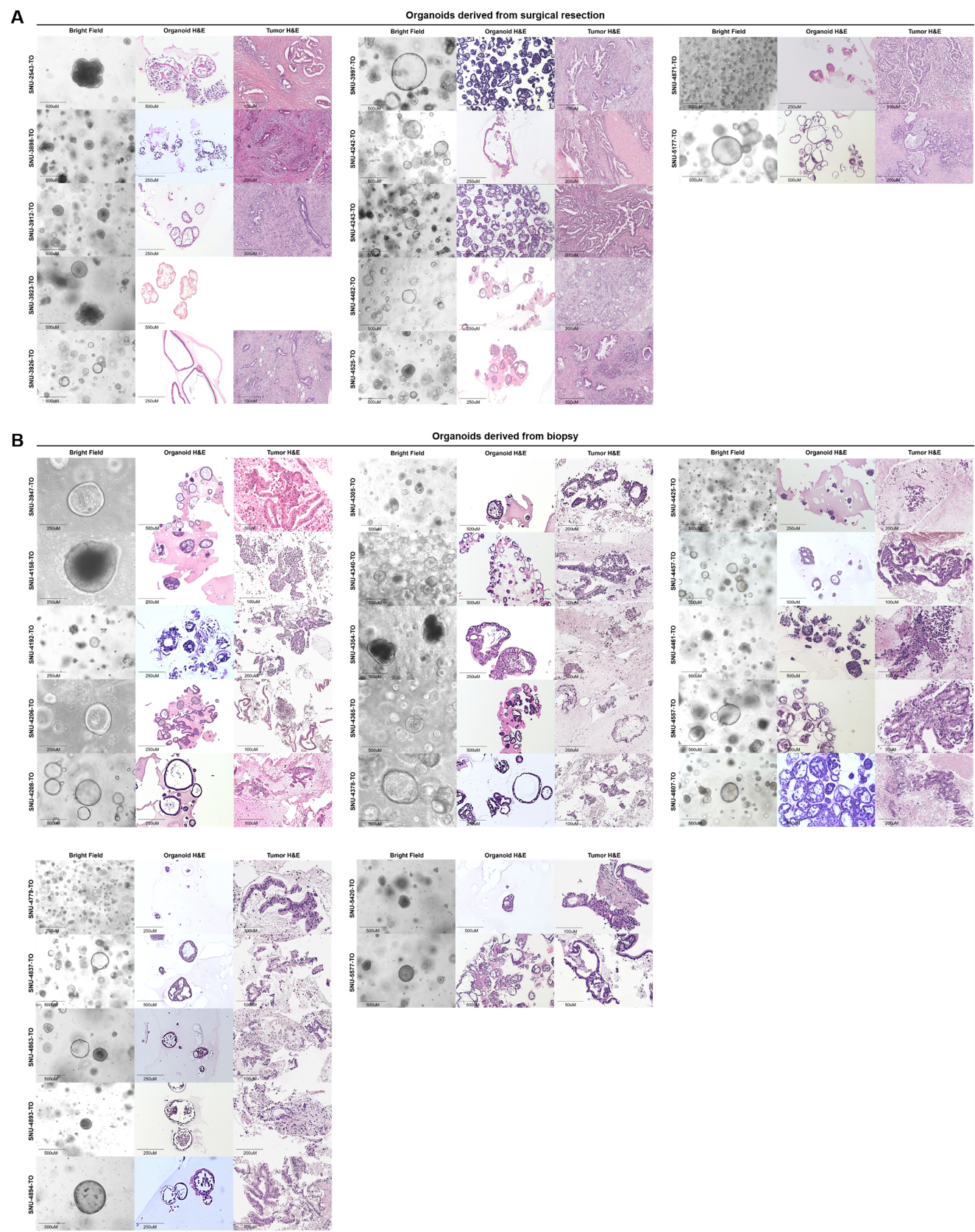


**Supplementary Fig.2.** The intensity of each color channel was analyzed. **2A.** The overall intensity of E-cadherin was normalized by dividing the intensity of E-cadherin expression (green channel) by the intensity of DAPI expression (blue channel). **2B.** The overall intensity of KI-67 was normalized by dividing the intensity of KI-67 expression (green channel) by the intensity of DAPI expression (blue channel).


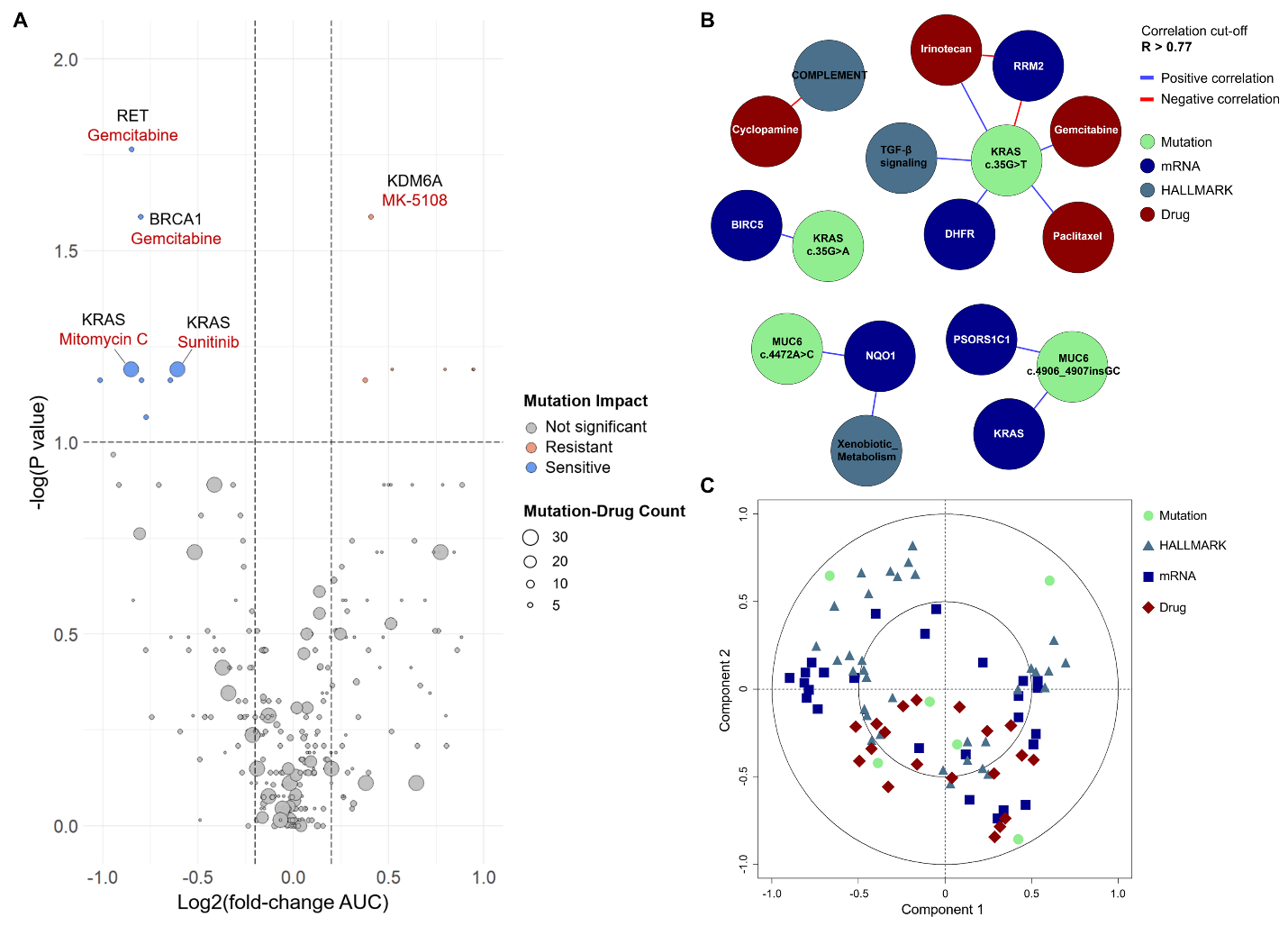


**Supplementary Fig. 3A.** The organoids retain majority of mutations in the original tumor tissues. Mutations were designated with shared, tissue-only and organoid only. Each mutation type was marked with representative colors. **3B.** Linear regression of variant allele frequency (VAF) of mutations within the tissue and organoid pairs was drawn. The Pearson correlation coefficient (R) with *p* value is designated above. **3C.** Local regression visualizing the dynamic pattern of variant allele frequencies (VAFs) between organoids and tissues. The Pearson correlation coefficient (R) with *p* value is designated above.


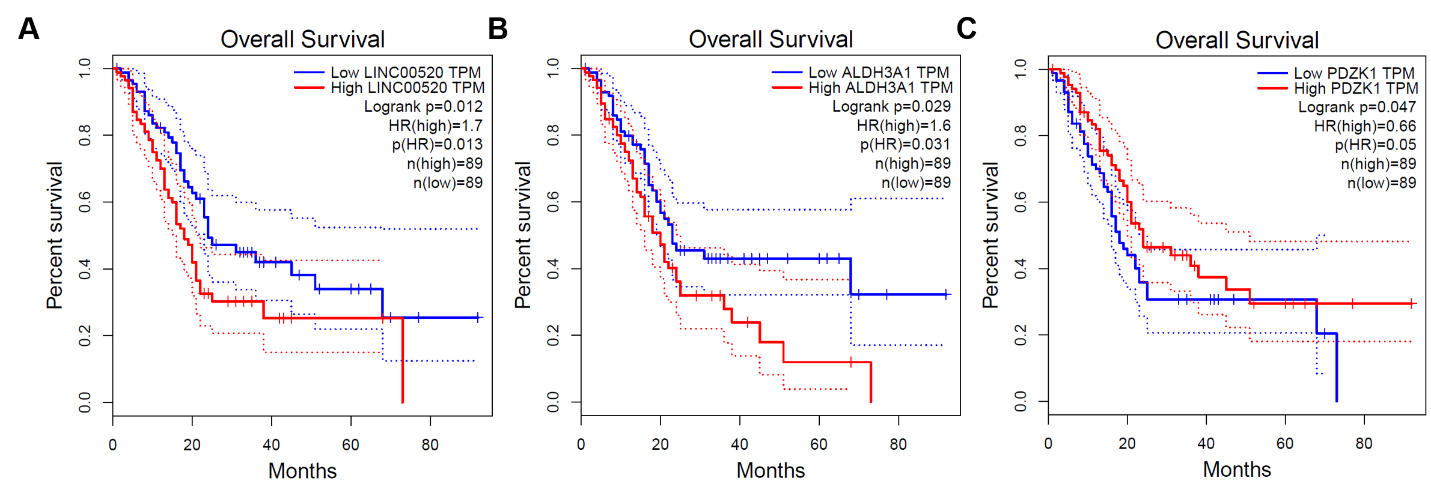


**Supplementary Fig. 4A.** Sixteen indel mutation types for both metastatic potential (MP) and non-metastatic potential (NMP) groups. The top facets display the types of indels, starting with C and T deletions, followed by C and T insertions, then multi-base deletions and insertions, and deletions associated with microhomology. The x-axis at the bottom indicates the number of repeat units. Each mutation type was specified with representative colors. **4B.** Mutational signature extracted from the relative point mutation types of our PDAC cohort revealed two characteristic signatures.


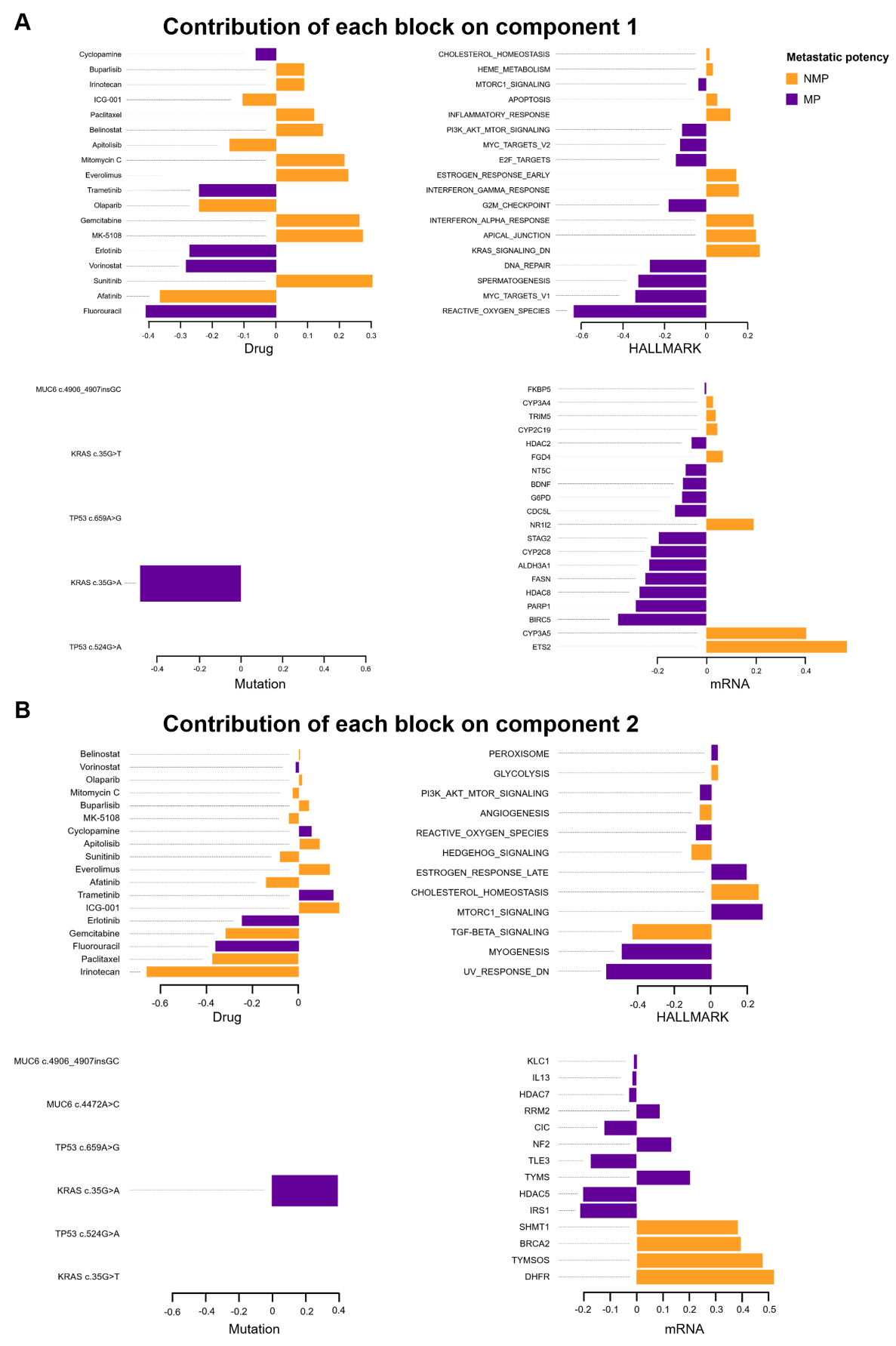


**Supplementary Fig. 5.** The overall survival is compared between low TPM group (Blue) and high TPM group (Red) for LINC00520 (**A**), ALDH3A1 (**B**), and PDZK1 (**C**) using TCGA-PAAD cohort. The hazard ratio with corresponding *p*-values were indicated top right.


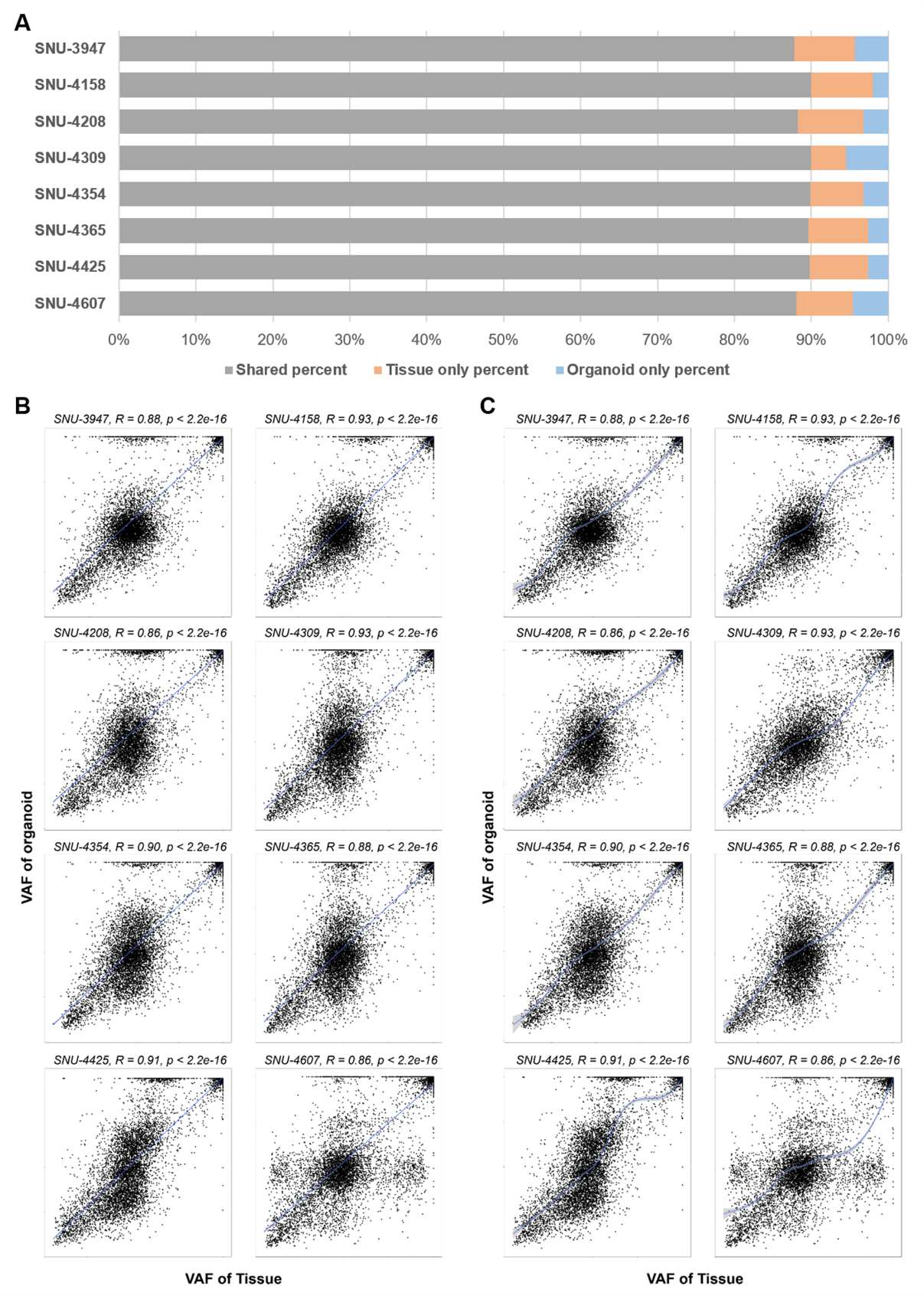


**Supplementary Fig. 6A.** Gene-drug interaction analysis using Wilcoxon rank sum test. Each dot indicated a pair of gene and drug. The size of the dot is proportional to the number of gene-drug set. When the mutational event was correlated to increased AUC values, the mutation was designated as resistant and colored in red. If the mutation was associated with decreased AUC values, it was assigned as sensitive and colored in blue. The absolute log fold change of AUC value > 0.2 and p <0.1 were considered as significant. **6B.** Multi-omics analysis identified multiple correlations (R > 0.77). Each block was marked with representative colors. **6C.** Principle analysis indicated the scattered pattern of each factor on two components. Each block was marked with representative colors.


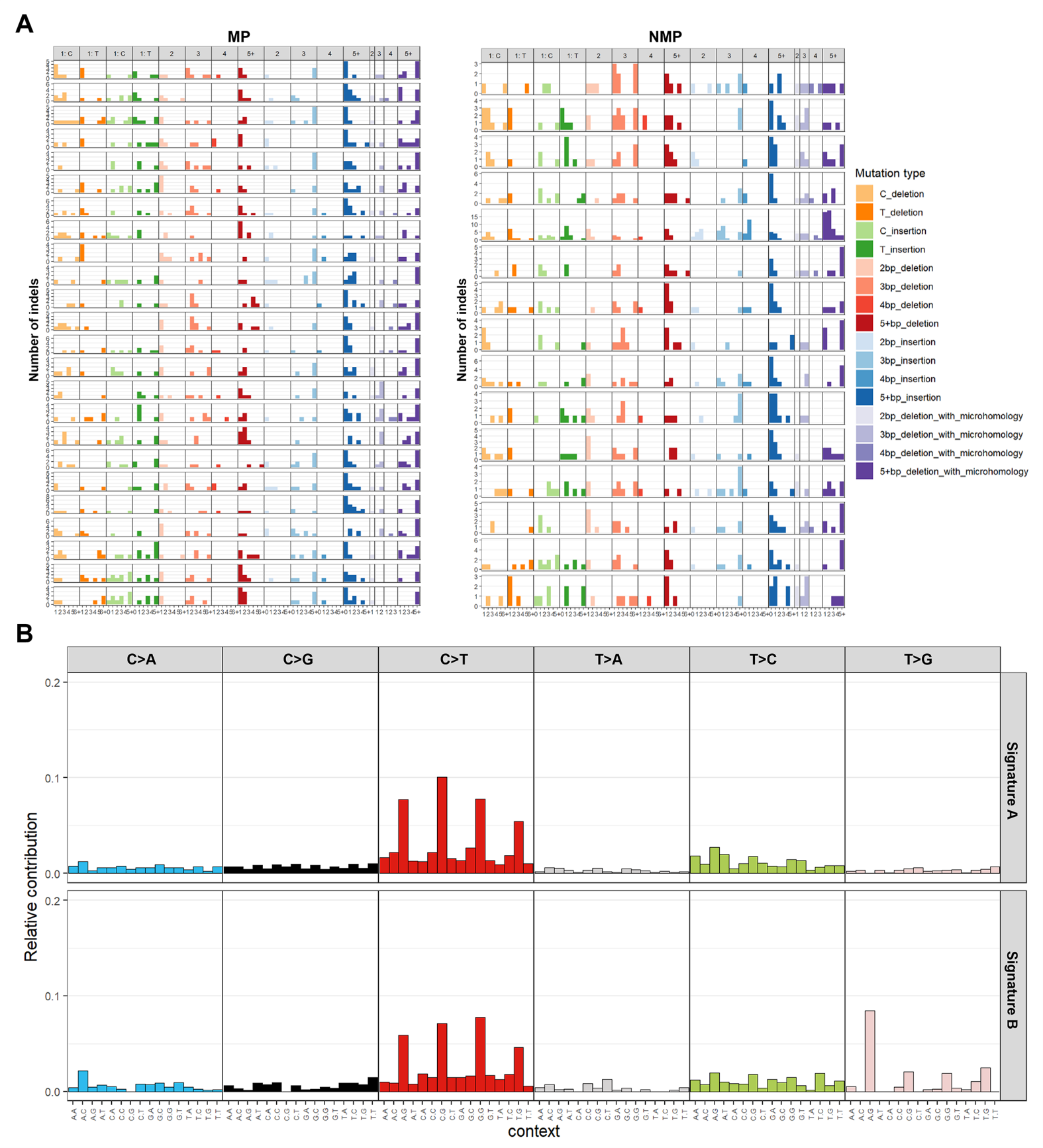
**Supplementary Fig. 7A.** Contribution of each block on component 1 is depicted in accordance with their metastatic potency. **7B.** Contribution of each block on component 2 is depicted in accordance with their metastatic potency.


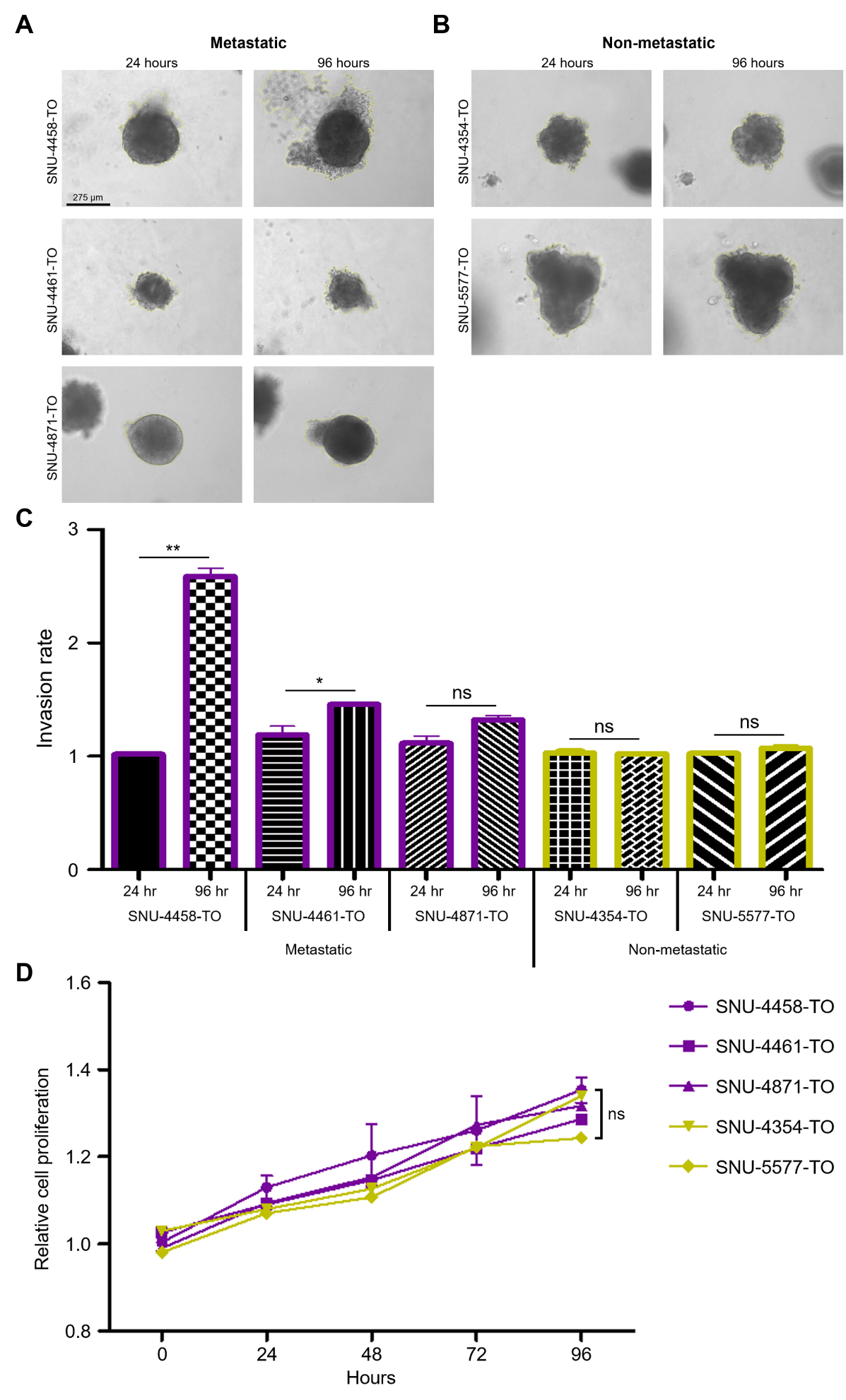


**Supplementary Fig. 8.** The invasion ability was compared between MP (metastatic potential) and NMP (non-metastatic potential) organoids. MP organoids exhibited higher invasiveness (**A**) compared to NMP organoids (**B**). The scale bar represents 250 μm. **8C.** Bar graph showed two MP organoids (SNU-4458-TO and SNU-4461-TO) invaded the invasion matrix significantly, while the other organoids showed lesser invasion. A P value < 0.05 was considered statistically significant (**, P < 0.01; *, P < 0.05; ns, P > 0.05). **8D.** Line graph exhibited that the proliferation rate of MP and NMP organoids was rarely different. The growth rate of SNU-4458-TO (MP, high cell proliferation rate) and SNU-5577-TO (NMP, low cell proliferation rate) 96 hours after initial seeding was compared, and there was no significant difference (ns, P > 0.05). (Purple; metastatic, Yellow; non-metastatic).


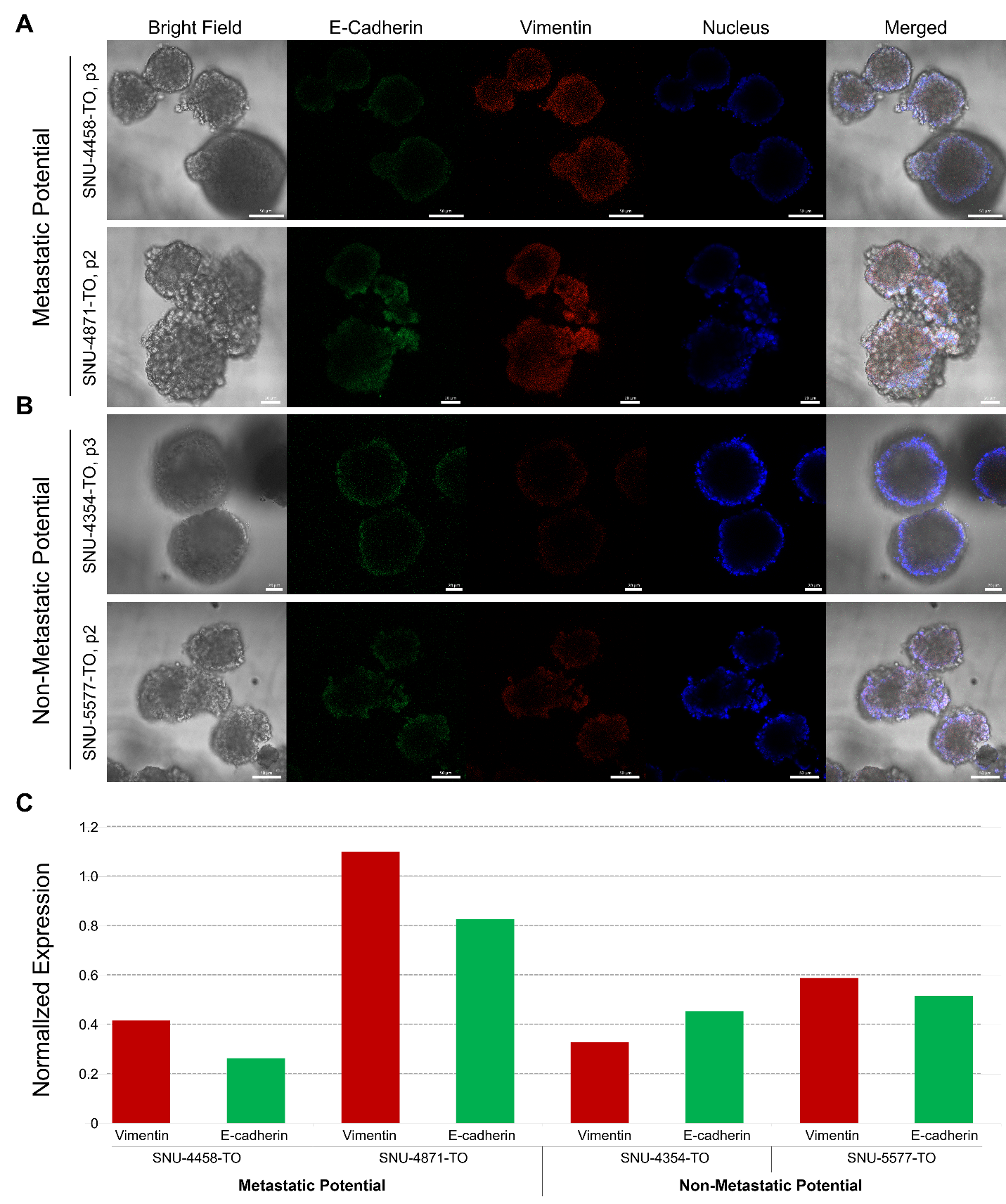


**Supplementary Fig. 9.** Expressional patterns of Vimentin (red channel) and E-cadherin (green channel) correlated with metastatic potential **(A)** and non-metastatic potential **(B)** are shown. The intensity of each signal is represented by a colored line graph. The scale bar is indicated at the bottom left side of the image. **9C.** The overall intensity of Vimentin (red channel) and E-cadherin (green channel) expression was normalized by dividing by the intensity of DAPI expression (blue channel).
